# Lysis reagents, cell numbers, and calculation method influence high-throughput measurement of HDL-mediated cholesterol efflux capacity

**DOI:** 10.1016/j.jlr.2021.100125

**Published:** 2021-09-25

**Authors:** Johanna F. Schachtl-Riess, Stefan Coassin, Claudia Lamina, Egon Demetz, Gertraud Streiter, Richard Hilbe, Florian Kronenberg

**Affiliations:** 1 Department of Genetics and Pharmacology, Institute of Genetic Epidemiology, Medical University of Innsbruck, Innsbruck, Austria; 2 Department of Internal Medicine II, Medical University of Innsbruck, Innsbruck, Austria

**Keywords:** HDL, macrophages, ABCA1, cholesterol/efflux, apolipoproteins, CEC assay, reverse cholesterol transport, HDL functionality, assay performance, CAVASIC, Cardiovascular Disease in Intermittent Claudication, CEC, cholesterol efflux capacity, CV, coefficient of variation, IQR, interquartile range, PEG, polyethylene glycol, RCT, reverse cholesterol transport, t0, time zero

## Abstract

HDL-mediated cholesterol efflux capacity (CEC) may protect against cardiovascular disease. However, CEC assays are not standardized, hampering their application in large cohorts and comparison between studies. To improve standardization, we systematically investigated technical differences between existing protocols that influence assay performance that have not been previously addressed. CEC was measured in 96-well plates using J774A.1 macrophages labeled with BODIPY-cholesterol and incubated for 4 h with 2% apolipoprotein B-depleted human serum. The time zero method, which calculates CEC using control wells, and the per-well method, which calculates CEC based on the actual content of BODIPY-cholesterol in each well, were compared in 506 samples. We showed that the per-well method had a considerably lower sample rejection rate (4.74% vs. 13.44%) and intra-assay (4.48% vs. 5.28%) and interassay coefficients of variation (two controls: 7.85%, 9.86% vs. 13.58%, 15.29%) compared with the time zero method. Correction for plate-to-plate differences using four controls on each plate also improved assay performance of both methods. In addition, we observed that the lysis reagent used had a significant effect. Compared with cholic acid, lysis with sodium hydroxide results in higher (*P* = 0.0082) and Triton X-100 in lower (*P* = 0.0028) CEC values. Furthermore, large cell seeding errors (30% variation) greatly biased CEC for both referencing methods (*P* < 0.0001) as measured by a resazurin assay. In conclusion, lysis reagents, cell numbers, and assay setup greatly impact the quality and reliability of CEC quantification and should be considered when this method is newly established in a laboratory.

HDL particles may protect form CVD because of their anti-inflammatory and antioxidative properties and because of their central role in macrophage reverse cholesterol transport (RCT) (1, 2). The RCT removes excess cholesterol from peripheral cells like macrophages and transports it to the liver for redistribution or biliary excretion. The cholesterol content of HDL particles (HDL-C) is a long-known predictor of CVD (3). However, Mendelian randomization studies and clinical trials of cholesteryl ester transfer protein inhibitors, which increase HDL-C concentrations, have indicated that HDL-C concentrations are not causally linked to CVD (4, 5). This suggests that the HDL-C content is a poor marker for HDL functionality.

The most studied function of HDL is its ability to remove cholesterol from macrophages, termed cholesterol efflux capacity (CEC). This is the initial step of RCT and can be measured in vitro with a cell-based assay (6, 7). Indeed, CEC is associated with incident and prevalent CVD (8, 9, 10, 11), but large studies in diverse populations are still sparse. Interestingly, in renal transplant recipients, CEC was not associated with CVD mortality but with graft failure, indicating that CEC might also play a role in other diseases (12, 13). To understand the role of CEC in CVD and also in other pathologies, more and large-scale studies are required.

Since the CEC assay is cell based, it is considered time intensive and cost intensive and hard to perform in a high-throughput setting. This has led to the development of novel methods evaluating HDL functionality in a cell-free environment (14, 15) or estimating CEC using LC-MS/MS techniques and nuclear magnetic resonance-based techniques (16, 17). These methods are a great tool to explore the association of CEC with outcomes in large general population studies. However, the estimated CEC used in these methods might not be a good predictor for CEC in other populations (16). Large studies using an actual cell-based CEC assay are thus still required to further explore the association of CEC with outcomes and to understand the genetic basis and regulation of CEC better (18, 19, 20).

The application of CEC assays to large studies is hampered by the fact that the assays are not well standardized, and various models have been used (21, 22). Hafiane and Genest (23) have previously addressed some aspects of the assay that can impact CEC results. In addition, recent large epidemiological studies agree in some technical key parameters (24, 25): cholesterol is labeled either radioactively with ^3^H or fluorescently with dipyrrometheneboron difluoride (BODIPY); J774A.1 macrophages or differentiated THP-1 cells are used; ABCA1 expression is upregulated by incubation with cAMP; apoB-depleted plasma or serum is used as acceptor; and efflux is most commonly performed for a time frame of 4 h. Nevertheless, there are still several technical differences between protocols (supplemental Tables S1 and S2). The impact of the cell type (26), label (7, 27), and acceptor (28, 29) on CEC has been discussed before (21). In contrast, the effect of different cell lysis reagents (7, 8, 9, 11, 12, 18) and the method for the calculation of the CEC (7, 9, 11, 26) have—to our knowledge—not been addressed but might be important for assay performance. In addition, no implementation of quality control measures for cell numbers and viability has been reported for the assay. We systematically tested the effect of these parameters on CEC results using BODIPY-labeled cholesterol. This system is mainly dependent on ABCA1-mediated CEC, which can be upregulated by cAMP, is saturable with increasing acceptor concentrations, and is associated with CVD in different cohorts (7, 9, 11, 30). We found that the type of lysis reagent and large differences in cell numbers indeed influence CEC measurements. Most importantly, the per-well method markedly improves assay performance compared with the time zero (t0) method.

## Materials and methods

### Samples

The protocol was established with sera from five healthy donors (three females and two males). Assay performance was investigated using serum samples from the Cardiovascular Disease in Intermittent Claudication (CAVASIC) study (31). All study participants provided written informed consent. The Ethical Committee of the participating study centers approved the examination protocol, and the study abides by the Declaration of Helsinki principles.

### CEC assay protocol

**Fig. 1:**
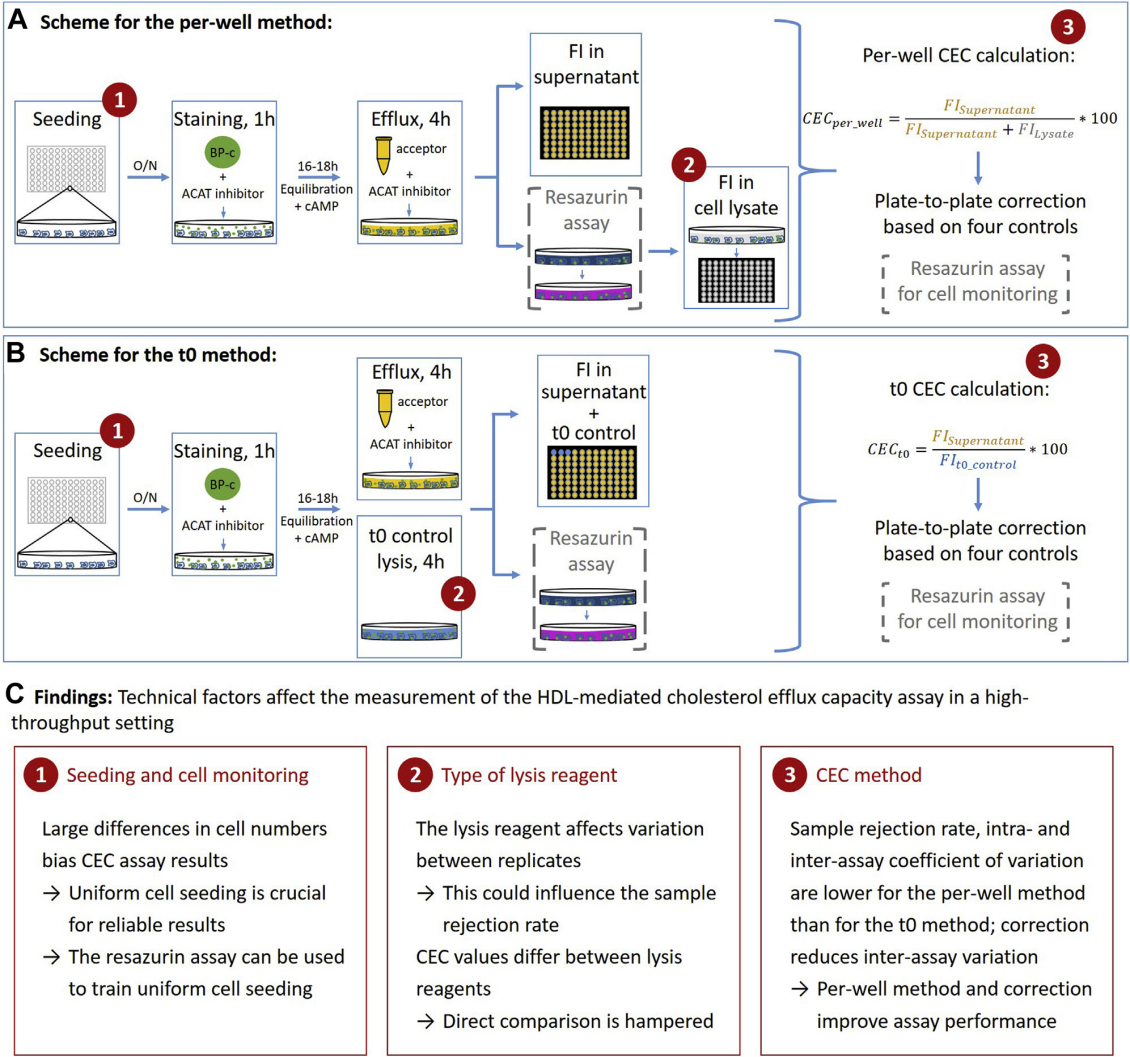
Overview of the evaluation process of the CEC protocol. Assay principle according to the (A) per-well method and (B) t0 method. The protocol is described in the Materials and Methods section, and a detailed protocol for the per-well method can be found in the Supplemental Methods section. Optionally, the resazurin assay can be implemented into the protocol for cell monitoring. The circled numbers indicate the steps where variations of the protocol have been compared. C: Summary of the comparisons. BP-c, BODIPY-cholesterol; FI, fluorescence intensity; O/N, overnight.

Figure 1 illustrates the protocol and which variations were investigated in this project. J774A.1 cells (ECACC 91051511; Sigma-Aldrich) were maintained in RPMI-1640 with phenol red and glutamine (Gibco) containing 10% fetal bovine serum (Gibco), 50 units/ml penicillin, and 50 μg/ml streptomycin (1× PenStrep; Gibco) at 37°C, and 5% CO_2_. For CEC measurements, cells were counted with a LUNA-FL dual fluorescence cell counter (Logos Biosystems). We seeded 7 × 10^4^ cells per well in 96-well plates and incubated them overnight (37°C, 5% CO_2_). All subsequent steps were performed in the presence of 2 μg/ml acetyl-CoA:cholesterol acyltransferase inhibitor (Sandoz 58-035; Sigma-Aldrich). Cells were stained for 1 h with 25 μM 23-(dipyrrometheneboron difluoride)-24-norcholesterol (9) (BODIPY-cholesterol; Avanti Polar Lipids) in phenol-red free RPMI-1640 (Gibco) containing 1× PenStrep, 1% fetal bovine serum, and 0.2% fatty acid-free bovine serum albumin (BioReagent). Cells were washed with Dulbecco's PBS containing Ca^2+^ and Mg^2+^ (Gibco) and equilibrated for 16–18 h in the presence of 0.3 mM 8-(4-chlorophenylthio)-cAMP sodium salt (Sigma-Aldrich).

Sera were stored at −80°C and thawed overnight on ice directly before use. ApoB depletion was done with 20% polyethylene glycol 6000 (PEG6000; in a.d.; Carl Roth). Immediately before efflux measurement, 15 μl of serum were mixed with 6 μl of 20% PEG6000, incubated (20 min, room temperature), and centrifuged (3,200 *g*, 30 min, 4°C). The apoB-depleted supernatant was taken for efflux measurement. Cells were incubated with 2% apoB-depleted sera (triplicates) for 4 h (37°C, 5% CO_2_). In three t0 control wells, instead of adding acceptor, cells were lysed with 1% cholic acid (cholic acid sodium salt; Carl Roth). The supernatant was centrifuged at 1,000 *g* for 15 min. Fluorescence intensity of BODIPY-cholesterol was measured at an excitation of 485 nm and an emission of 530 nm with a SPARK microplate reader (Tecan) at optimal gain.

Subsequently, cells were washed and lysed with three different lysis reagents: *i*) 1% cholic acid; *ii*) 0.1 M sodium hydroxide, or *iii*) 1% Triton X-100 at 1,200 rpm at room temperature for 1 h. Fluorescence intensity in the cell lysates was measured with the same settings and gain as the supernatant.

A more detailed protocol is available in the Supplemental Data.

### CEC according to t0 method and per-well method

**Fig. 2:**
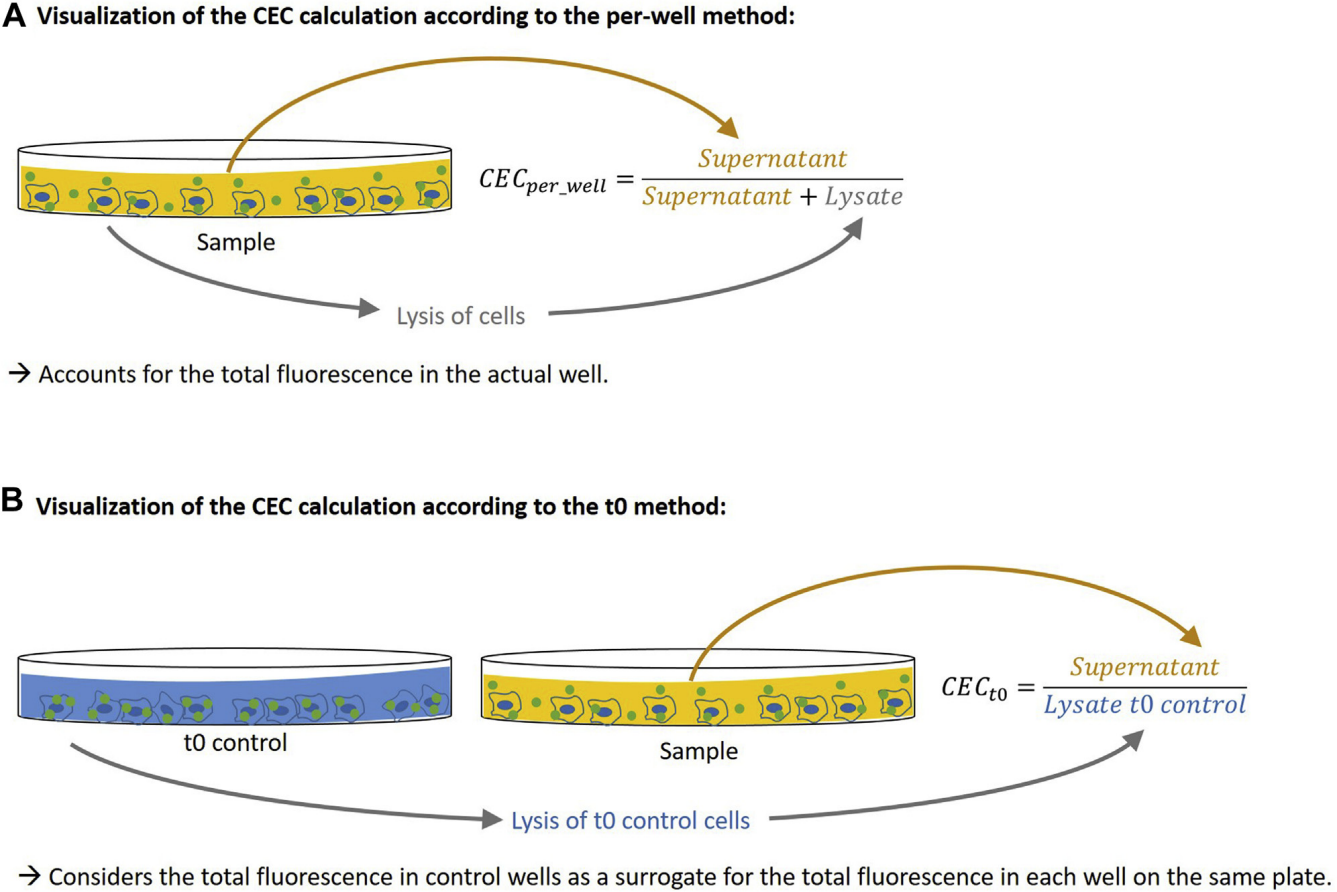
Visualization of the CEC calculation according to the (A) per-well method and (B) t0 method. Compared with the t0 method, the per-well method in addition requires the lysis of cells of all samples (Fig. 1). However, the advantage is that it accounts for the total fluorescence in the actual well instead of using a control as a surrogate. The detailed calculations can be found in the Materials and Methods section.

CEC was calculated referencing either to (Equation 1) the total fluorescence of each well (CEC_per_well_) (26) or (Equation 2) the t0 control wells (CEC_t0_) (7) (Fig. 2). In either case, first the background fluorescence of supernatant or cell lysate from unstained cells was subtracted from the fluorescence of the supernatant or the cell lysate. CEC_per_well_ was calculated as

(1) $$CEC_{per\text{\_}well}=\frac{FI_{Sup}}{FI_{Sup}+FI_{Lys}}\times 100$$

FI_Sup_ denotes the fluorescence of the supernatant from a sample after efflux, and FI_Lys_ is the fluorescence of the cell lysate of the corresponding well.

CEC_t0_ was calculated as

(2) $$CEC_{t\text{0}}=\frac{FI_{Sup}}{FI_{t\text{0\_}control}}\times 100$$

FI_Sup_ is the fluorescence of the supernatant of a sample after efflux to the acceptor (i.e., apoB-depleted serum). FI_t0_control_ is the fluorescence of wells that were lysed at t0 of the efflux by addition of lysis reagent instead of adding acceptor. Subsequently, passive efflux (to medium without acceptor) was subtracted from the CEC values of both methods.

The sample rejection rate (excluded or repeated samples because of a coefficient of variation [CV] of replicates >15%) is defined as rejected samples divided by total samples.

Intra-assay CV in CAVASIC study was calculated as the mean CV of replicates of all samples that were not rejected. Interassay CV was calculated based on the CEC values of two controls across all plates (n = 25).

To correct for plate-to-plate differences, four additional control samples (that were not used for interassay CV monitoring) were assessed on each plate. The relative difference of expected and real CEC values was calculated for each control. The mean of the relative difference of the four controls was used as correction factor. The interassay CVs before and after correction were routinely compared using two additional controls not used for correction.

### Introduction of the resazurin assay into the CEC assay

The resazurin assay (Canvax) was implemented into the CEC assay to monitor the cells after taking the supernatant for fluorescence measurement (Fig. 1A, B). After washing, instead of lysing cells directly, 100 μl of 10% resazurin solution in phenol red-free RPMI-1640 (1× PenStrep and 0.2% fatty acid free bovine serum albumin) was added to the cells. Cells were incubated (37°C, 5% CO_2_, 3 h), and absorbance was measured at 570 and 600 nm with a SPARK reader. Subsequently, the medium containing resazurin was discarded, cells were washed twice, and lysed as described in the aforementioned CEC assay protocol.

The absorbance ratio at 570/600 nm was calculated, and the absorbance ratio of a well without cells was subtracted as blank value.

### CEC assay with varying cell numbers

To test in a controlled experiment whether systematic cell seeding errors bias the results of the CEC assay, we seeded 0.5, 0.75, 1, and 1.5 times the standard cell amount in triplicates (3.5 × 10^4^, 5.25 × 10^4^, 7 × 10^4^, and 1.05 × 10^5^ cells/well), and the CEC assay protocol including the resazurin assay was performed.

### Statistical analysis

All analyses were performed using R, version 3.6.1 (R Core Team) and RStudio, version 1.2.1335 (R Studio PBC). The correlation of the resazurin assay with the actual number of cells was tested by Pearson correlation. Linear mixed models were used to assess differences and effect sizes of measurements repeated with varying conditions (lysis reagent, cell numbers, and cAMP treatment) (nlme package (https://cran.r-project.org/package=nlme, accessed November 10, 2020)). Bland-Altman plots were used to test agreement between repeated measurements (BlandAltmanLeh package (https://cran.r-project.org/package=BlandAltmanLeh, accessed November 13, 2020)). Data were fitted to the Michaelis-Menten equation (drc package (32)) as described previously (7) to model the effect of increasing amounts of apoB-depleted serum on CEC.

## Results

Considerable technical differences exist between reported CEC assay protocols (supplemental Tables S1 and S2). We therefore tested the effect of different lysis reagents, cell numbers, and assay setups on the CEC assay results. These results are provided in the following subchapters. A summary is given in Fig. 1.

### Comparison of lysis reagents

**Fig. 3:**
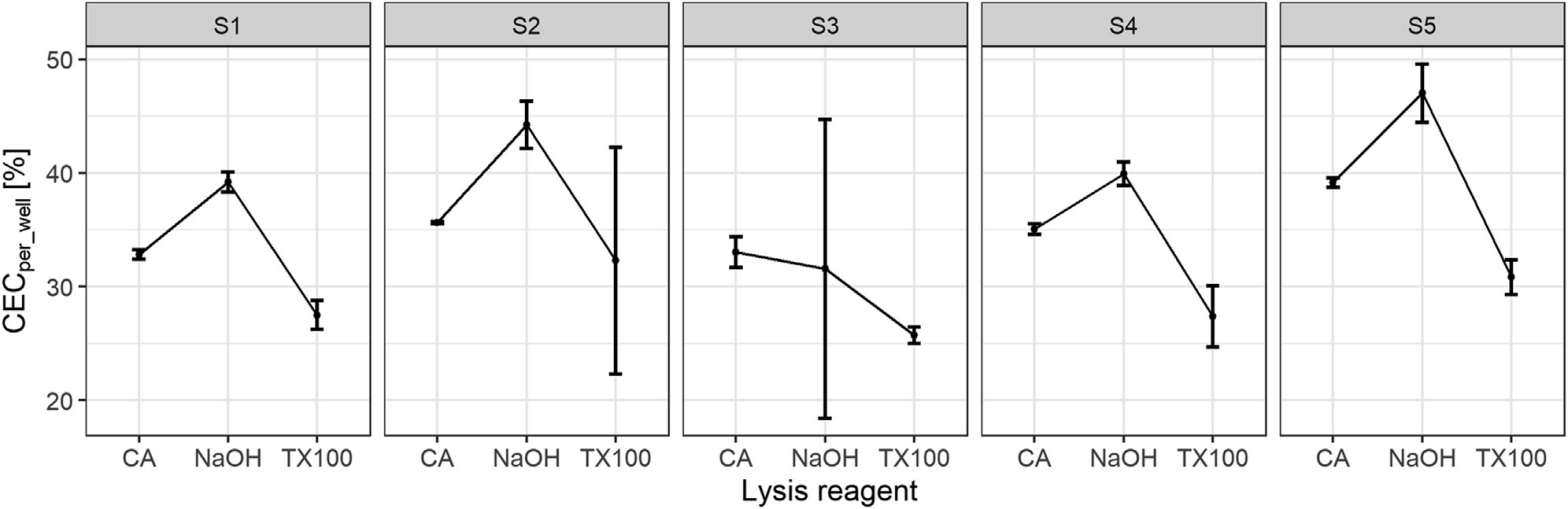
Effect of the lysis reagent on CEC. Compared with cholic acid, lysis with sodium hydroxide results in significantly higher CEC values (*P* = 0.0082) and lysis with TX100 in significantly lower CEC values (*P* = 0.0028). The SD between replicates is lowest for lysis with cholic acid. Dots and error bars represent mean and SD of triplicates. If no error bars are visible, they are contained within the symbol because of a very small variability of the measurements. CEC was calculated per well. CA, cholic acid; NaOH, sodium hydroxide; S1–5, sample 1–5; TX100, Triton X-100.

The CEC of five healthy donors was measured using cholic acid, sodium hydroxide, or Triton X-100 for lysis. These reagents have been used in published CEC protocols and can easily be used in high-throughput studies (supplemental Table S2) (7, 12) or are likely the active component of commercial CEC kits (Abcam; ab196985). The lysis reagent affected both the CEC values of the samples and their variance. The median CV was 1% for cholic acid and 5% for sodium hydroxide and Triton X-100. Compared with the percent of CEC using cholic acid, the measured CEC was higher by 5.3% using sodium hydroxide as lysis reagent (*P* = 0.0082) and lower by 6.4% using Triton X-100 (*P* = 0.0028). This corresponds to a 1.15- and a 0.82-fold change in CEC (Fig. 3). Assessment of lysis completeness showed that the fluorescence remaining in the wells was low for cholic acid and Triton X-100 (2.4% [interquartile range (IQR): 1.7–3.0] and 1.9% [IQR: 1.6–2.4], respectively), whereas it was 6.1% (IQR: 4.9–6.8) for sodium hydroxide, likely explaining the higher CEC values with sodium hydroxide (supplemental Fig. S1). Given the lower variation between replicates for cholic acid as lysis reagent, all subsequent experiments were done using cholic acid as lysis reagent.

### Effect of different cell numbers on CEC

The resazurin assay is a high-throughput capable colorimetric assay measuring the metabolic activity of cells. This is quantified by the reduction of resazurin into resorufin, which is proportional to the cell number (supplemental Fig. S2) (33). We thus introduced the resazurin assay into the CEC protocol to monitor cell numbers and seeding homogeneity during the CEC assay.

**Fig. 4:**
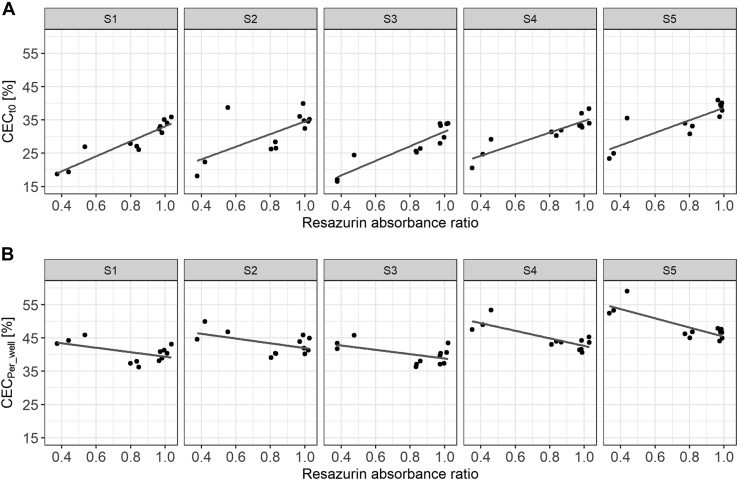
Large differences in cell numbers bias CEC assay results. CEC measured with the (A) t0 method and (B) per-well method dependent on the resazurin absorbance ratio. Each dot represents a single measurement of the CEC and resazurin assay (four different cell numbers plated in triplicates). Lines represent the linear regression line of the respective sample. S1–5, samples 1–5.

To assess the effect of different cell numbers, we performed an experiment with a large and deliberate seeding error resulting in a CV of the resazurin assay (used as proxy for cell numbers) of 29.67%. Measurement of the CEC using both methods (t0 and per-well methods) showed that cell numbers significantly biased CEC results (Fig. 4). While the CEC measured using the t0 method increased on average by 4.75% CEC (95% CI: 3.90, 5.59; *P* < 0.0001) per 1 SD increase of the resazurin absorbance ratio, the CEC measured using the per-well method decreased on average by 2.23% CEC (95% CI: −2.93; −1.53; *P* < 0.0001). Furthermore, significantly, more cells were dead if 0.5 times or 1.5 times of the standard cell number were seeded per well (supplemental Fig. S3).

### Comparison of assay performance using the t0 method and per-well method

The CEC can be measured with two different setups: (Equation 1) per-well and (Equation 2) using t0 controls (Fig. 2). In the per-well method, the results for each well are referenced to the total fluorescence of the individual well. The t0 method uses as control a triplicate estimate per plate of the total fluorescence that should be contained in every well. Both setups replicated the known assay features of cAMP dependency (supplemental Figs. S4 and S5) and saturability of the assay with increasing acceptor concentration following a Michaelis-Menten model (supplemental Figs. S6 and S7).

To compare the impact of the two methods, we performed the CEC assay in 506 samples from the CAVASIC study and calculated both CEC values. Each sample was measured in triplicates. The per-well method showed a 65% lower sample rejection rate (samples with an intra-assay CV >15%) than the t0 method (Table 1). The intra-assay CV was comparable albeit 15% lower for the per-well method (4.48% vs. 5.28%; Table 1). Also, the interassay CV was lower for both CEC controls for the per-well method (Table 1). Of note, we also observed that training and experience in handling the assay influence assay performance: when we measured CEC with the per-well method afterward in another large study, only 1.4% of samples needed to be repeated because of a CV above 15% (vs. 4.7% in the present study).

**Table 1:** Assay performance of the t0 and per-well method

Assay performance characteristics	t0 Method	Per-Well Method	Relative Reduction by Per-Well Method
Sample rejection rate (n rejected/n total)	13.44% (68/506)	4.74% (24/506)	65%
Intra-assay CV^a^	5.28%	4.48%	15%
Uncorrected interassay CV for PC1	16.74%	11.88%	29%
Uncorrected interassay CV for PC2	16.34%	11.55%	29%
Corrected interassay CV for PC1^b^	13.58%	7.85%	42%
Corrected interassay CV for PC2^b^	15.29%	9.86%	36%
Relative reduction interassay CV for PC1 by correction^b^	19%	34%	—
Relative reduction interassay CV for PC2 by correction^b^	6%	15%	—

PC, positive controls consist of two samples (PC1 and PC2).

a Excluding rejected samples.

b Corrected for interassay differences using four additional controls as described in the Materials and Methods section.

**Fig. 5:**
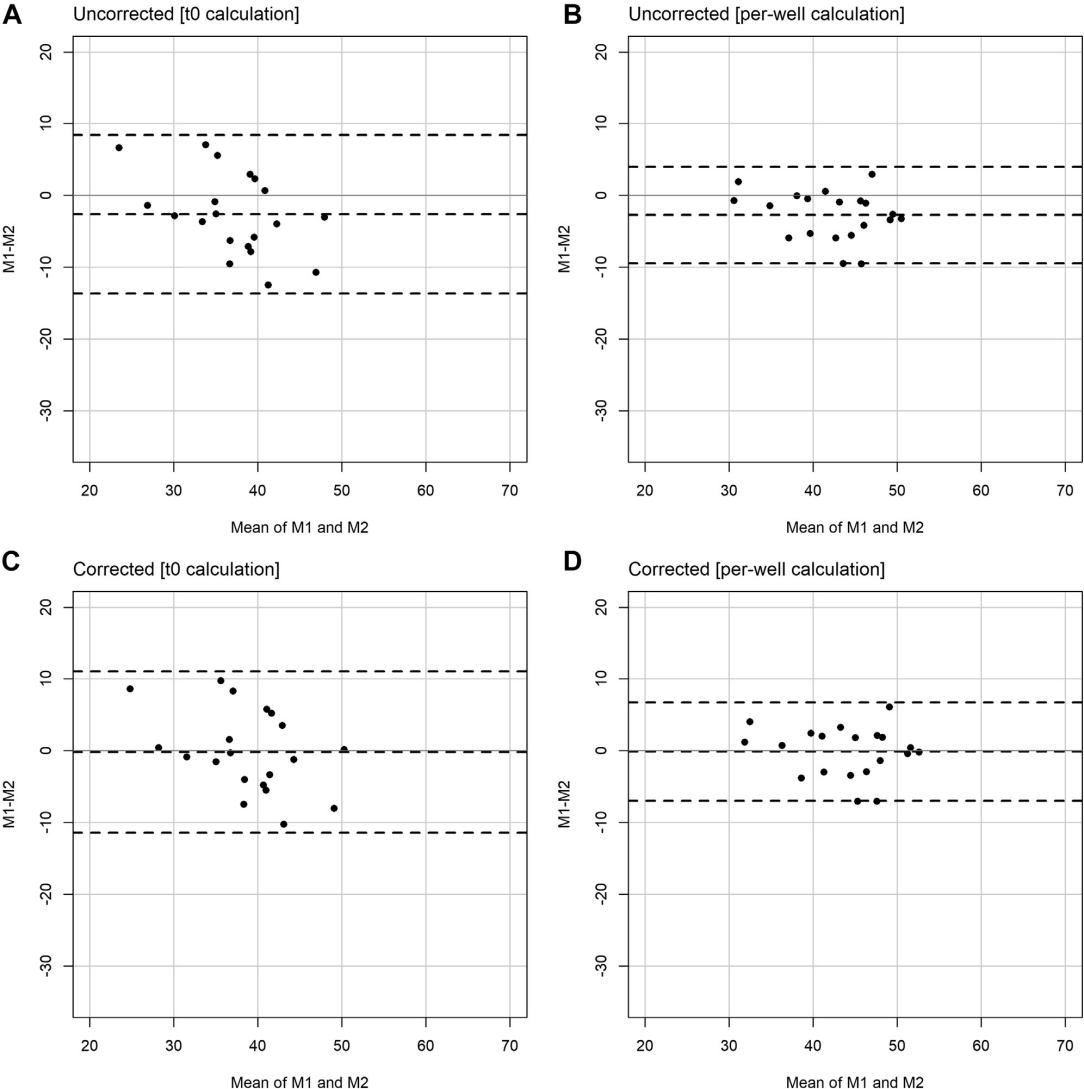
Bland-Altman plots for CEC measured twice 25 days apart. A: Uncorrected measurements for the t0 method. B: Uncorrected measurements for the per-well method. C: Corrected measurements for the t0 method. D: Corrected measurements for the per-well method. In total, 28 samples were measured twice, but eight samples had to be rejected in the t0 method because of high CV of replicates. For better comparability, these samples have also been excluded from the plots for the per-well method. The plots including the rejected samples are shown in supplemental Fig. S8. Dashed lines represent mean ± 2 SDs of differences (95% limits of agreement). The gray solid line represents difference = 0. M1, measurement 1; M2, measurement 2.

Interassay differences can bias the CEC assay (Fig. 5A, B). We therefore used a plate correction factor calculated from four additional controls to correct for plate-to-plate differences as described in the Materials and Methods section. These controls were not used for interassay CV monitoring and were measured on each plate in the same way as the other samples. This reduced the interassay CV (Table 1). Most importantly, it also reduced the average bias between measurements repeated 25 days apart from −2.64% to −0.19% CEC (92.9%) for the t0 method and from −2.74% to −0.15% CEC (94.4%) for the per-well method (Fig. 5). Of note, the 95% limits of agreement of the per-well method are narrower than of the t0 method, indicating higher accuracy (Fig. 5).

As shown in Fig. 4, large systematic seeding errors can bias CEC assay results. To test if seeding errors are a problem in a standardized setting, we monitored cell numbers by resazurin assay during CEC measurement of CAVASIC study. Differences in cell numbers were very low with an intra-assay and interassay CV for the resazurin assay of 3.28 and 9.13%, respectively.

To assess whether normalization to the cell number reduces intra-assay and interassay CV of the CEC assay, we normalized the results of both methods to the resazurin absorbance ratio. While intra-assay and interassay CV for the t0 method did not differ to a relevant extent between resazurin-normalized and non-normalized data, resazurin normalization increased intra-assay and interassay CV of the per-well method (supplemental Table S3) (addressed in the Discussion section).

## Discussion

There is probably no lipoprotein that has raised more controversies during the last decade than HDL. This might be caused by the many faces of HDL particles with its hundreds of proteins and lipid species (34), which tempted us to compare it with a chameleon (13, 35). Cell-based assays that investigate the functionality of HDL particles on the one hand might bring us closer to a better understanding but are on the other hand hard to standardize to improve comparability of study results. In addition, the increased interest for CEC and the need to analyze it in large populations is bringing new research groups into the field. These face the challenge to set up the assay in a standardized and reproducible manner. While general methodological descriptions of the assay exist, challenges that are relevant in high-throughput settings have not been addressed yet. For example, small but systematic errors can lead to significant bias and spurious results. The intent of this study was to systematically evaluate technical aspects of the fluorescently labeled CEC assay in a high-throughput setting to lay the basis for an improvement of the assay standardization. We show that lysis reagent, cell number, and the referencing method as well as the plate-to-plate correction affect CEC assay performance. In summary, the best assay performance was observed with the combination of lysis with cholic acid, CEC measurement per-well method, and plate-to-plate correction using four control samples. The use of the resazurin assay may be helpful for general cell monitoring but not for normalization. This setup also replicates cAMP dependency and saturation with increasing acceptor concentration (7), indicating its overall validity. A step-by-step protocol of this setup is provided in the Supplemental Methods section.

We observed that the CV between CEC replicates was lower when cholic acid was used as a lysis reagent compared with sodium hydroxide and Triton X-100. This higher CV of replicates could increase the sample rejection rate (samples with a high CV) in a study setting. In addition, the measured CEC values were 1.15-fold higher for lysis with sodium hydroxide and were 0.82-fold lower for lysis with Triton X-100 compared with lysis with cholic acid. This hampers direct comparison of CEC values that were measured using different lysis reagents and highlights the importance of reporting the type of lysis reagent. Of note, hexane:isopropanol has been used as lysis reagent in radioactive protocols (supplemental Table S1) but has health and environmental issues and is highly volatile. Since volatility is especially problematic when working with small volumes as it is the case in a high-throughput assay, it can confound fluorescence measurements (36). Therefore, we would not use it as lysis reagent for CEC assays using BODIPY-cholesterol.

Reproducible and uniform cell seeding is required for cell-based assays. We show that 30% variation in the resazurin absorbance ratio (used as a proxy for cell numbers) significantly biases CEC assay results, irrespective of the measurement method. However, this bias was smaller for the per-well method than for the t0 method (−2.23 vs. +4.75 effect size on CEC per 1 SD increase of the absorbance ratio). Notably, there was a positive bias for increasing absorbance ratios for the t0 method, whereas there was a negative bias for the per-well method. This could be explained by their technical basis. The t0 method uses a control to estimate the total BODIPY-cholesterol that should be contained in every well. The BODIPY-cholesterol in the t0 control is invariable, whereas the amount in other wells increases with increasing cell numbers. Consequently, more BODIPY-cholesterol can be released into the supernatant in wells containing more cells leading to a higher calculated CEC. This problem is absent for the per-well method since the actual content of BODIPY-cholesterol in every well is considered. However, efflux from cAMP-treated J774A.1 macrophages increases with increasing amounts of acceptor (supplemental Fig. S6 (6, 7)). When the number of cells per well increases, the ratio of acceptor to cells decreases likely leading to the observed negative association of resazurin assay with CEC. The per-well method may thus implicitly account for small variations in cell numbers and staining efficiencies but is biased if the ratio of acceptor to number of cells changes markedly. In addition, other factors such as more dead cells if the cell layer is not in a confluent state (supplemental Fig. S3) could also impact CEC, further highlighting the importance of uniform and optimal cell seeding.

Furthermore, we monitored cell numbers with the resazurin assay while measuring CEC in a standardized setting with uniform cell seeding in 506 samples. Variation was very low (intra-assay and interassay CV of 3.28 and 9.13%, respectively). Resazurin normalization of CEC values did not markedly improve assay performance for the t0 method, indicating that the variance of this method is not explained by this small variation of cell numbers. In contrast, normalization even increased the interassay CV for the per-well method. The observation that the per-well method already accounts for small variations in cell numbers may explain why normalizing to this variation again via the resazurin normalization could increase variation. Overall, we did not find evidence that resazurin normalization provides additional benefit beyond standard quality control measures like regular visual inspection of cells in the microscope and exclusion of samples with overly high variation. However, uniform seeding is crucial for unbiased CEC results, highlighting the importance of good training prior to conducting CEC assays. The resazurin assay is a valuable tool to control cell numbers and avoid systematic seeding errors, especially during assay establishment.

In 506 serum samples, the assay performance of the per-well method was better than of the t0 method (Table 1). First, the sample rejection rate for the per-well method was 65% lower, which is especially relevant in high-throughput settings and reduces working time, cost, usage, and freeze-thaw cycles of valuable study samples. Second, the intra-assay CVs (5.3% for the t0 method and 4.5% for the per-well method) are comparable to the intra-assay CVs reported in the literature for the t0 method (3.3–7.1% (9, 37)) and the per-well method (3.1–6.2% (18, 26)). Third, our direct comparison of the two methods shows that the interassay CV of the per-well method (7.85/9.86%) is on average 39% lower than of the t0 method (13.58/15.29%) (Table 1). The reported interassay CV in the literature for the t0 method is 6.3–9.0% (8, 19), whereas it is 7.6–18.4% (18, 26) for the per-well method. However, the protocols differ between the publications. The better performance of the per-well method is also expected from a technical point since it internally controls for the total fluorescence in each well (Fig. 2). Of note, the per-well method has also been used in earlier experiments (termed as fractional efflux) (38). To conclude, our direct comparison of the two methods, which have both been used in large studies before (supplemental Tables S1 and S2), indicates that the per-well method is superior in terms of efficiency (less samples to repeat; Table 1) and accuracy (lower interassay CV and 95% limits of agreement; Table 1 and Fig. 5).

To correct for interassay differences, CEC values are usually normalized to one value (pooled control) (8, 9). In contrast and as a strength of our protocol, we used a similar approach but calculated a plate-to-plate correction factor based on four different control samples. This is more robust if one of the controls deviates from its expected value because of technical variation in the individual sample not reflecting an overall difference of the CEC of all samples on the plate. In addition, it leaves the individual CEC values on their original scale (i.e., percent of total BODIPY-cholesterol and not CEC [sample] relative to CEC [control]), easing comparison between studies. Unfortunately, it is rarely reported in the literature whether plate-to-plate correction improved assay performance. Low-Kam *et al.* (18) reported that correction reduced the global CV by >3%, which is similar to the reductions that we observed in our experiment (relative reduction of >6% for t0 method and >15% for per-well method; Table 1). Importantly, we observed that the introduction of four control samples for the plate-to-plate correction massively reduced the bias of CEC measured twice 25 days apart for both methods (by 92.9% for the t0 method and by 94.4% for the per-well method).

In the study at hand, we systematically investigate the effect of the lysis reagent, cell numbers, and referencing method on CEC using BODIPY-labeled cholesterol. We acknowledge that additional differences between CEC protocols like the type of cholesterol label, cell line, and acceptor have an impact on CEC but have been intensively investigated before (7, 9, 21, 23, 26, 28, 29, 39, 40). A limitation is that we did not test the protocol in cholesterol-loaded cells. While cholesterol loading may influence overall assay performance, it is unlikely to change the effect of different lysis reagents, cell numbers, and methods to control for the variability of CEC on assay performance compared with each other. The exact experimental design also depends on the research question, but the overlap between protocols from large studies may help to guide the choice of the general assay setup (e.g., cell type, delivery of labeled cholesterol, and type of acceptor). Of note, many previous CEC studies used ^3^H-labeled cholesterol, which, however, poses obvious difficulties. Therefore, BODIPY-cholesterol has been proposed recently as an alternative that is easier to use in high-throughput settings (7). Studies have confirmed that also CEC using BODIPY-cholesterol is associated with CVD in different cohorts (9, 11, 30).

To better understand the true role of CEC in health and disease, we have to bite the bullet and measure cell-based CEC in large populations. In the study at hand, we show that lysis reagent, cell number, and methods to control for variability of CEC are critical technical aspects that influence assay performance and thus the quality of results. These should be considered when setting up the CEC assay. It further highlights the importance of constantly monitoring quality control measures, such as sample rejection rate, intra-assay and interassay CV, and reduction of interassay CV after correction.

## Data availability

Data are available from the corresponding author on reasonable request.

## Supplemental data

This article contains supplemental data (8, 9, 10, 11, 12, 18, 19, 20, 24, 25, 26, 30, 37, 41, 42, 43, 44, 45, 46, 47, 48, 49).

## Conflict of interest

The authors declare that they have no conflicts of interest with the contents of this article.

## References

[1] Karathanasis S.K., Freeman L.A., Gordon S.M., Remaley A.T. (2017). The changing face of HDL and the best way to measure it. Clin. Chem..

[2] Lewis G.F., Rader D.J. (2005). New insights into the regulation of HDL metabolism and reverse cholesterol transport. Circ. Res..

[3] Gordon T., Castelli W.P., Hjortland M.C., Kannel W.B., Dawber T.R. (1977). High density lipoprotein as a protective factor against coronary heart disease. The Framingham study. Am. J. Med..

[4] Voight B.F., Peloso G.M., Orho-Melander M., Frikke-Schmidt R., Barbalic M., Jensen M.K., Hindy G., Hólm H., Ding E.L., Johnson T., Schunkert H., Samani N.J., Clarke R., Hopewell J.C., Thompson J.F. (2012). Plasma HDL cholesterol and risk of myocardial infarction: a mendelian randomisation study. Lancet.

[5] Tall A.R., Rader D.J. (2018). Trials and tribulations of CETP inhibitors. Circ. Res..

[6] De La Llera-Moya M., Drazul-Schrader D., Asztalos B.F., Cuchel M., Rader D.J., Rothblat G.H. (2010). The ability to promote efflux via ABCA1 determines the capacity of serum specimens with similar high-density lipoprotein cholesterol to remove cholesterol from macrophages. Arterioscler. Thromb. Vasc. Biol..

[7] Sankaranarayanan S., Kellner-Weibel G., de la Llera-Moya M., Phillips M.C., Asztalos B.F., Bittman R., Rothblat G.H. (2011). A sensitive assay for ABCA1-mediated cholesterol efflux using BODIPY-cholesterol. J. Lipid Res..

[8] Khera A.V., Cuchel M., de la Llera-Moya M., Rodrigues A., Burke M.F., Jafri K., French B.C., Phillips J.A., Mucksavage M.L., Wilensky R.L., Mohler E.R., Rothblat G.H., Rader D.J., Ph D., Phillips J.A. (2011). Cholesterol efflux capacity, high-density lipoprotein function, and atherosclerosis. N. Engl. J. Med..

[9] Rohatgi A., Khera A., Berry J.D., Givens E.G., Ayers C.R., Wedin K.E., Neeland I.J., Yuhanna I.S., Rader D.R., de Lemos J.A., Shaul P.W. (2014). HDL cholesterol efflux capacity and incident cardiovascular events. N. Engl. J. Med..

[10] Saleheen D., Scott R., Javad S., Zhao W., Rodrigues A., Picataggi A., Lukmanova D., Mucksavage M.L., Luben R., Billheimer J., Kastelein J.J.P., Boekholdt S.M., Khaw K.-T., Wareham N., Rader D.J. (2015). Association of HDL cholesterol efflux capacity with incident coronary heart disease events: a prospective case-control study. Lancet Diabetes Endocrinol..

[11] Liu C., Zhang Y., Ding D., Li X., Yang Y., Li Q., Zheng Y., Wang D., Ling W. (2016). Cholesterol efflux capacity is an independent predictor of all-cause and cardiovascular mortality in patients with coronary artery disease: a prospective cohort study. Atherosclerosis.

[12] Annema W., Dikkers A., Freark de Boer J., Dullaart R.P.F., Sanders J.-S.F., Bakker S.J.L., Tietge U.J.F. (2016). HDL cholesterol efflux predicts graft failure in renal transplant recipients. J. Am. Soc. Nephrol..

[13] Kronenberg F. (2018). High-density lipoprotein in chronic kidney diseases—the devil is in the detail. J. Am. Soc. Nephrol..

[14] Lorkowski S.W., Brubaker G., Li L., Li X.S., Hazen S.L., Smith J.D. (2020). A novel cell-free fluorescent assay for HDL function: low apolipoprotein A1 exchange rate associated with increased incident cardiovascular events. J. Appl. Lab. Med..

[15] Harada A., Toh R., Murakami K., Kiriyama M., Yoshikawa K., Miwa K., Kubo T., Irino Y., Mori K., Tanaka N., Nishimura K., Ishida T., Hirata K. (2017). Cholesterol uptake capacity: a new measure of HDL functionality for coronary risk assessment. J. Appl. Lab. Med..

[16] Kuusisto S., Holmes M.V., Ohukainen P., Kangas A.J., Karsikas M., Tiainen M., Perola M., Salomaa V., Kettunen J., Ala-Korpela M. (2019). Direct estimation of HDL-mediated cholesterol efflux capacity from serum. Clin. Chem..

[17] Jin Z., Collier T.S., Dai D.L.Y., Chen V., Hollander Z., Ng R.T., McManus B.M., Balshaw R., Apostolidou S., Penn M.S., Bystrom C. (2019). Development and validation of apolipoprotein AI-associated lipoprotein proteome panel for the prediction of cholesterol efflux capacity and coronary artery disease. Clin. Chem..

[18] Low-Kam C., Rhainds D., Lo K.S., Barhdadi A., Boulé M., Alem S., Pedneault-Gagnon V., Rhéaume E., Dubé M., Busseuil D., Hegele R.A., Lettre G., Tardif J. (2018). Variants at the APOE /C1/C2/C4 locus modulate cholesterol efflux capacity independently of high-density lipoprotein cholesterol. J. Am. Heart Assoc..

[19] Koekemoer A.L., Codd V., Masca N.G.D., Nelson C.P., Musameh M.D., Kaess B.M., Hengstenberg C., Rader D.J., Samani N.J. (2017). Large-scale analysis of determinants, stability, and heritability of high-density lipoprotein cholesterol efflux capacity. Arterioscler. Thromb. Vasc. Biol..

[20] Ritsch A., Duerr A., Kahler P., Hunjadi M., Stojakovic T., Silbernagel G., Scharnagl H., Kleber M.E., März W. (2020). Cholesterol efflux capacity and cardiovascular disease: the Ludwigshafen Risk and Cardiovascular Health (LURIC) study. Biomedicines.

[21] Rhainds D., Tardif J.-C.C. (2019). From HDL-cholesterol to HDL-function. Curr. Opin. Lipidol..

[22] Anastasius M., Kockx M., Jessup W., Sullivan D., Rye K.A., Kritharides L. (2016). Cholesterol efflux capacity: an introduction for clinicians. Am. Heart J..

[23] Hafiane A., Genest J. (2015). HDL-mediated cellular cholesterol efflux assay method. Ann. Clin. Lab. Sci..

[24] Qiu C., Zhao X., Zhou Q., Zhang Z. (2017). High-density lipoprotein cholesterol efflux capacity is inversely associated with cardiovascular risk: a systematic review and meta-analysis. Lipids Health Dis..

[25] Soria-Florido M.T., Schröder H., Grau M., Fitó M., Lassale C. (2020). High density lipoprotein functionality and cardiovascular events and mortality: a systematic review and meta-analysis. Atherosclerosis.

[26] Li X.M., Tang W.H.W., Mosior M.K., Huang Y., Wu Y., Matter W., Gao V., Schmitt D., DiDonato J.A., Fisher E.A., Smith J.D., Hazen S.L. (2013). Paradoxical association of enhanced cholesterol efflux with increased incident cardiovascular risks. Arterioscler. Thromb. Vasc. Biol..

[27] Ritsch A., Scharnagl H., März W. (2015). HDL cholesterol efflux capacity and cardiovascular events. Eur. Heart J..

[28] Davidson W.S., Heink A., Sexmith H., Melchior J.T., Gordon S.M., Kuklenyik Z., Woollett L., Barr J.R., Jones J.I., Toth C.A., Shah A.S. (2016). The effects of apolipoprotein B depletion on HDL subspecies composition and function. J. Lipid Res..

[29] Holzer M., Kern S., Trieb M., Trakaki A., Marsche G. (2017). HDL structure and function is profoundly affected when stored frozen in the absence of cryoprotectants. J. Lipid Res..

[30] Tejera-Segura B., Macía-Díaz M., Machado J.D., de Vera-González A., García-Dopico J.A., Olmos J.M., Hernández J.L., Díaz-González F., González-Gay M.A., Ferraz-Amaro I. (2017). HDL cholesterol efflux capacity in rheumatoid arthritis patients: contributing factors and relationship with subclinical atherosclerosis. Arthritis Res. Ther..

[31] Kheirkhah A., Lamina C., Rantner B., Kollerits B., Stadler M., Pohlhammer J., Klein-Weigel P., Fraedrich G., Kronenberg F. (2021). Elevated levels of serum PCSK9 in male patients with symptomatic peripheral artery disease: the CAVASIC study. Atherosclerosis.

[32] Ritz C., Baty F., Streibig J.C., Gerhard D. (2015). Dose-response analysis using R. PLoS One.

[33] Riss T.L., Moravec R.A., Niles A.L., Duellman S., Benink H.A., Worzella T.J., Minor L., Markossian S., Grossman A., Brimacombe K., Arkin M., Auld D., Austin C.P., Baell J., Chung T.D.Y., Coussens N.P., Dahlin J.L., Devanarayan V., Foley T.L., Glicksman M., Hall M.D., Haas J.V. (2004).

[34] Annema W., von Eckardstein A. (2016). Dysfunctional high-density lipoproteins in coronary heart disease: implications for diagnostics and therapy. Transl. Res..

[35] Kronenberg F. (2011). Genetic variation in HDL-related genes and the association with cardiovascular disease: HDL particles as chameleons of lipoprotein metabolism. J. Intern. Med..

[36] Cottingham M.G., Bain C.D., Vaux D.J.T. (2004). Rapid method for measurement of surface tension in multiwell plates. Lab. Investig..

[37] Potočnjak I., Degoricija V., Trbušić M., Terešak S.D., Radulović B., Pregartner G., Berghold A., Tiran B., Marsche G., Frank S. (2016). Metrics of high-density lipoprotein function and hospital mortality in acute heart failure patients. PLoS One.

[38] De la Llera Moya M., Atger V., Paul J.L., Fournier N., Moatti N., Giral P., Friday K.E., Rothblat G. (1994). A cell culture system for screening human serum for ability to promote cellular cholesterol efflux: relations between serum components and efflux, esterification, and transfer. Arterioscler. Thromb..

[39] Low H., Hoang A., Sviridov D. (2012). Cholesterol efflux assay. J. Vis. Exp..

[40] Adorni M.P., Zimetti F., Billheimer J.T., Wang N., Rader D.J., Phillips M.C., Rothblat G.H. (2007). The roles of different pathways in the release of cholesterol from macrophages. J. Lipid Res..

[41] Annema W., Willemsen H.M., de Boer J.F., Dikkers A., van der Giet M., Nieuwland W., Muller Kobold A.C., van Pelt L.J., Slart R. H.J.A., van der Horst I.C.C., Dullaart R.P.F., Tio R.A. (2016). HDL function is impaired in acute myocardial infarction independent of plasma HDL cholesterol levels. J. Clin. Lipidol..

[42] Bauer L., Kern S., Rogacev K.S., Emrich I.E., Zawada A., Fliser D., Heinemann A., Heine G.H. (2017). HDL cholesterol efflux capacity and cardiovascular events in patients with chronic kidney disease. J. Am. Coll. Cardiol..

[43] Hunjadi M., Lamina C., Kahler P., Bernscherer T., Viikari J., Lehtimäki T., Kähönen M., Hurme M., Juonala M., Taittonen L., Laitinen T., Jokinen E., Tossavainen P., Hutri-Kähönen N., Raitakari O. (2020). HDL cholesterol efflux capacity is inversely associated with subclinical cardiovascular risk markers in young adults: The cardiovascular risk in Young Finns study. Sci. Rep..

[44] Ishikawa T., Ayaori M., Uto-Kondo H., Nakajima T., Mutoh M. (2015). High-density lipoprotein cholesterol efflux capacity as a relevant predictor of atherosclerotic coronary disease. Atherosclerosis.

[45] Khera A.V., Demler O.V., Adelman S.J., Collins H.L., Glynn R.J., Ridker P.M., Rader D.J. (2017). Cholesterol efflux capacity, high-density lipoprotein particle number, and incident cardiovascular events: an analysis from the JUPITER Trial (Justification for the Use of Statins in Prevention: An Intervention Trial Evaluating Rosuvastatin). Circulation.

[46] Kopecky C., Ebtehaj S., Genser B., Drechsler C., Krane V., Antlanger M., Kovarik J.J., Kaltenecker C.C., Parvizi M., Wanner C., Weichhart T., Säemann M.D. (2017). HDL cholesterol efflux does not predict cardiovascular risk in hemodialysis patients. J. Am. Soc. Nephrol..

[47] Ogura M., Hori M. (2016). Association between cholesterol efflux capacity and atherosclerotic cardiovascular disease in patients with familial hypercholesterolemia. Arterioscler. Thromb. Vasc. Biol..

[48] Shea S., Stein J.H., Jorgensen N.W., McClelland R.L., Tascau L., Shrager S., Heinecke J.W., Yvan-Charvet L. (2019). Cholesterol mass efflux capacity, incident cardiovascular disease, and progression of carotid plaque: the multi-ethnic study of atherosclerosis. Arterioscler. Thromb. Vasc. Biol..

[49] Zhang J., Xu J., Wang J., Wu C., Xu Y., Wang Y., Deng F., Wang Z., Chen X., Wu M. (2016). Prognostic usefulness of serum cholesterol efflux capacity in patients with coronary artery disease. Am. J. Cardiol..

