# Supplementary material for: Lysis reagents, cell numbers, and calculation method influence high-throughput measurement of HDL-mediated cholesterol efflux capacity
Source: J Lipid Res. 2021 Sep 25;62:100125. doi: 10.1016/j.jlr.2021.100125 (PMC8521207; doi:10.1016/j.jlr.2021.100125)
Supplement: Supplemental Methods, Tables S1–S3 and Figures S1–S8 [file mmc1.pdf]

Supplemental Data to

Lysis reagents, cell numbers, and calculation method influence high-throughput measurement of HDL-mediated cholesterol efflux capacity

Johanna F. Schachtl-Riess [1], Stefan Coassin [1], Claudia Lamina [1], Egon Demetz [2], Gertraud Streiter [1], Richard Hilbe [2], Florian Kronenberg [1]

[1] Institute of Genetic Epidemiology, Department of Genetics and Pharmacology, Medical University of Innsbruck, Innsbruck, Austria

[2] Department of Internal Medicine II, Medical University of Innsbruck, Innsbruck, Austria

Corresponding author:
Florian Kronenberg, MD
Institute of Genetic Epidemiology
Department of Genetics and Pharmacology
Medical University of Innsbruck
Schöpfstrasse 41, A-6020 Innsbruck, Austria.
Phone: (+43) 512-9003-70560
Fax: (+43) 512 9003-73560 or -73561,
Email: Florian.Kronenberg@i-med.ac.at

Content

Content 1
Supplemental Methods 2
Detailed CEC assay protocol for the per-well method 2
Media 2
Protocol 2
Comments 4
Supplemental Table S1: Comparison of CEC assay protocols in different studies. 7
Supplemental Table S2: Summary of the previously published CEC assay protocols listed in Supplemental Table S1. 8
Supplemental Table S3: Comparison of assay performance of CEC values normalized to the resazurin absorbance ratio and non-normalized CEC values. 9
Supplemental Figure S1: Fluorescence remaining in the plate after lysis for 1 hour and overnight for cholic acid (CA), sodium hydroxide (NaOH) and Triton-X-100 (TX100). 10
Supplemental Figure S2: Correlation of cell number with the resazurin absorbance ratio. 11
Supplemental Figure S3: Viability differs between seeded cell numbers. 12
Supplemental Figure S4: For the per-well method, CEC from cAMP-treated cells is significantly higher than CEC from untreated cells (p=0.0006, n=5). 13
Supplemental Figure S5: For the t0 method, CEC from cAMP-treated cells is significantly higher than CEC from untreated cells (p=0.0015, n=5). 14
Supplemental Figure S6: CEC saturation curves for increasing amounts of apoB-depleted serum of five healthy individuals for the per-well method. 15
Supplemental Figure S7: CEC saturation curves for increasing amounts of apoB-depleted serum of five healthy individuals for the t0 method. 16
Supplemental Figure S8: Bland-Altman plots for CEC measured twice 25 days apart including samples with high CV. 17
References 18

Supplemental Methods

Detailed CEC assay protocol for the per-well method

Media

Culture medium

- RPMI-1640 with phenol red and glutamine
- 10% FBS
- 50 units/mL penicillin and 50 µg/mL streptomycin (1x PenStrep)

Staining medium

- RPMI-1640 with glutamine, phenol red-free
- 25 µM BODIPY-cholesterol [(i)]
- 2 µg/mL ACAT inhibitor (Sandoz 58-035)
  (stored in stock solutions of 5000 µg/mL in DMSO at -20°C) [(ii)]
- 1x PenStrep
- 1% FBS
- 0.2% fatty acid free BSA (20% stock in a.d., sterile filtered and stored at -20 °C) [(ii)]

Equilibration medium

- RPMI-1640 with glutamine, phenol red-free
- 0.3 mM 8-CPT-cAMP (stock 30 mM in a.d., sterile filtered and stored at -20 °C) [(ii)]
- 2 µg/mL ACAT inhibitor (Sandoz 58-035)
  (stored in stock solutions of 5000 µg/mL in DMSO at -20°C) [(ii)]
- 1x PenStrep
- 0.2% fatty acid free BSA (20% stock in a.d., sterile filtered and stored at -20 °C) [(ii)]

Efflux medium

- RPMI-1640 with glutamine, phenol red-free
- 2 µg/mL ACAT inhibitor (Sandoz 58-035)
  (stored in stock solutions of 5000 µg/mL in DMSO at -20°C) [(ii)]

Protocol

A schematic overview of the assay principle is provided in Figure 1A. J774A.1 cells are maintained in culture medium at 37 °C, 5% CO_2[(iii)]. For CEC measurements, $7x10^4$ cells per-well are seeded in tissue culture-treated 96-well plates in 100 µL of culture medium and incubated over night at 37 °C, 5% CO_2[(iv)]. The next day, culture medium is removed and cells are stained for one hour in 100 µL of staining medium[(v)]. Please note that in two to three control wells, cells

should not be stained to be able to determine background fluorescence of supernatant and cell lysate later on. Afterwards, cells are washed 1x with 200 µL DPBS containing Ca^{2+} and Mg^{2+} and equilibrated for 16-18 hours in 100 µL of equilibration medium containing cAMP to upregulate expression of ABCA1.

Efflux is performed using apoB-depleted sera. ApoB-depleted sera are prepared from sera by precipitation with 20% PEG6000 (in a.d.) directly before efflux measurement. Sera should be stored at -80 °C and thawed on ice prior to depletion. For apoB depletion, 10 parts of serum (15 µL) are mixed with 4 parts of 20% PEG6000 (6 µL), incubated for 20 minutes at room temperature, centrifuged at 3200 g for 30 min at 4 °C and supernatant is taken for efflux measurement.

After removing the equilibration medium and washing cells 1x with 200 µL DPBS containing Ca^{2+} and Mg^{2+}, cells are incubated with 110 µL of efflux medium containing 2% apoB-depleted serum for four hours at 37 °C and 5% CO_2. Please note that no apoB-depleted serum should be contained in the efflux medium in 1) the background fluorescence controls described in the staining step above and 2) two to three control wells that will be used to calculate the passive efflux. Then, 100 µL of the supernatant is transferred to conically-shaped wells (V-bottom) and centrifuged at 1000 g for 15 min at room temperature to remove cells[(vi)]. Subsequently, 80 µL of the supernatant is transferred to a black assay plate[(vii)]. Fluorescence intensity (FI) of BODIPY-cholesterol is measured at excitation 485 nm and emission 530 nm with a multimode microplate reader[(viii)].

After removal of the supernatant from the tissue culture treated plates for the FI measurement in the step above, the cells are washed 1x with 200 µL DPBS containing Ca^{2+} and Mg^{2+} and then cells are lysed with 110 µL of 1% cholic acid (in a.d.) at 1200 rpm at room temperature for 1 hour. Afterwards, 80 µL of cell lysate is transferred to a black assay plate and FI of BODIPY-cholesterol in the cell lysates is measured with the same settings and gain as FI of the supernatant[(viii)].

CEC is calculated per-well. First, background fluorescence of supernatant or cell lysate from unstained cells is subtracted from the FI value for supernatant or cell lysate, respectively. Then, CEC is calculated as $CEC_{per\_well} = \frac{FI_{Sup}}{FI_{Sup}+FI_{Lys}} * 100$. FI_{Sup} is the FI of the supernatant from a sample after efflux to the added acceptor and FI_{Lys} is the FI of the cell lysate from the

corresponding well after efflux. Subsequently, passive efflux (to medium without acceptor and calculated in the same way as CEC of a sample) is subtracted[ix].

We measured CEC in duplicates or triplicates. If CV of replicates is above 15%, we repeat or exclude these samples. To be able to monitor inter-assay differences, we include a high- and low positive control on every plate. Additionally, we correct for inter-assay differences with four controls which are included on every plate. To calculate an inter-assay correction factor for every plate, the relative difference of expected (mean over the whole measurement series) and real CEC value is calculated for each control. The mean of the four controls is used as correction factor. To monitor if correction for inter-assay differences reduces the inter-assay CV, inter-assay CV before and after inter-assay correction are compared[x].

Comments

i) BODIPY-cholesterol stock is stored at -20 °C at a concentration of 867.58 µM in 100% EtOH in aliquots. Due to the low temperature during freezing, BODIPY-cholesterol precipitates. To dissolve the precipitated BODIPY-cholesterol in EtOH, the solution is sonicated for 10 min at 37 °C directly before usage, spun down to pellet still undissolved BODIPY-cholesterol and then supernatant is added to warm staining medium. We do not recommend to lower the concentration of the BODIPY-cholesterol stock since this would increase the proportion of EtOH – which is toxic for cells – in the staining medium.

ii) All reagents which are stored at -20°C are stored in aliquots. Aliquots are never refrozen and are used within 24 hours after thawing.

iii) General cell culture rules apply: Regularly check for mycoplasma contamination, ensure uniformity in cell growth and split cells in exponential growth phase.

iv) Ensure that passages of cells do not differ too much, we only use cells for efflux which are maximum 10 passages apart. Additionally, as described in the results, seeding errors can bias CEC assay results. Thus, we strongly recommend to train even and reproducible seeding using the resazurin assay or another cell monitoring assay prior to conducting the CEC assay. From our experience, seeding works best by seeding a maximum of 24 wells at a time with a multi-stepper pipette. Always gently mix your stock cell solution directly before taking cells for seeding with the multi-stepper pipette.

v) We recommend to use 8-channel pipettes and an 8-channel vacuum pump for all processing steps to avoid drying-out of cells. In addition, supernatant should always be removed or taken for further processing from the side of the well to avoid damaging the cell layer. If available, some steps could also be performed with liquid handling robots.

vi) Since some cells are floating in the supernatant, this step is crucial to improve precision and reproducibility. The supernatant could also be filtered, however we compared different centrifugation and filtering protocols (data not shown) and got the highest reproducibility of results using this protocol.

vii) The excess volume during incubation (110 µL for efflux, 100 µL for spinning down and 80 µL for FI measurement) ensures equal volumes in all reactions for the FI measurement, which is essential for a high reproducibility. The volumes for the cholic acid during lysis have to be the same for FI to be comparable. The volume for the FI measurement should not be reduced further since then the meniscus of the liquid in the well and the lower depth of the liquid could falsify the measurement.

viii) As suggested by the manufacturers of multimode microplate readers, the FI of the supernatant should be measured at the optimal gain of the photomultiplier tube (PMT). Usually, the readers can perform an automatic gain adjustment before the measurement. To calculate the CEC per-well with the FI of the supernatant and the cell lysate of the same well (which are measured on two separate plates) it is critical that the FI of both supernatant and cell lysate are measured at the same gain. Thus, the gain in the FI measurement of the cell lysate should be manually set to the gain that was used to measure the FI of the supernatant. To avoid saturation of the signals when measuring the FI of the lysate, we recommend to add one control well to the FI measurement of the supernatant in which the cells are lysed to avoid high automatic gain values which can cause saturation of the FI signals of the cell lysates. In short this means:

   a) To avoid saturation of FI signals in step b) include a control well on every plate where stained cells are lysed with 1% cholic acid (i.e. instead of adding 110 µL efflux medium in this control well, the same amount of 1% cholic acid is added and processed exactly as the efflux media in the rest of the plate). Measure FI of supernatant at optimal gain.

   b) Measure FI of cell lysates at the same gain as the supernatant in step a).

ix) FI of supernatant and lysate from unstained cells have to be subtracted because the background fluorescence of medium and 1% cholic acid differs. To calculate the efflux that is specific for the acceptor (in this case 2% apoB-depleted serum), the efflux without an acceptor (passive efflux) needs to be subtracted.

x) In summary this means that the following controls should be included on every plate:

   a) Unstained cells for subtraction of the background FI

   b) Stained cells that are lysed to set the optimal gain during FI measurement of the supernatant

   c) Stained cells that are incubated with efflux medium without acceptor to calculate the passive efflux

   d) Control samples (in total we use six control samples: four for inter-assay cor and two for monitoring the inter assay CV)

Supplemental Table S1: Comparison of CEC assay protocols in different studies.

Studies were selected if they were included in two recent meta-analysis(1, 2), performed a cell-based CEC protocol in >100 individuals and were the first description of a study population. In addition, the studies of Hunjadi et al., 2020, Koekemoer et al., 2017, Low-Kam et al., 2018 and Ritsch et al., 2020 were added manually since they performed a cell-based CEC protocol in >1,000 individuals.

Study	Size	Cell line	Label	cAMP	Acceptor[a]	Duration of efflux	Measurement method	Lysis reagent	Intra-assay CV	Inter-assay CV	Reduction inter-assay CV
**Annema et al., 2016**(3)	495	THP-1	3H	-	plasma	6 hours	per-well	NaOH	NA	NA	NA
**Annema et al., 2016**(4)	110	THP-1	3H	-	plasma	24 hours	per-well	NaOH	5.4%	NA	NA
**Bauer et al., 2017**(5)	526	J774	3H	NA	serum	NA	NA	NA	7.3%	6.9%	NA
**Hunjadi et al., 2020**(6)	2282	J774	3H	yes	serum	4 hours	relative to control	-	NA	NA	NA
**Ishikawa et al., 2015**(7)	254	J774	3H	no	serum or plasma	4 hours	t0	hex isoprop	NA	NA	NA
**Khera et al., 2011**(8)	996	J774	3H	yes	serum	4 hours	t0	hex isoprop	5%	9%	NA
**Khera et al., 2017**(9)	628	J773	3H	yes	plasma	NA	NA	NA	3.7%	4.7%	NA
**Tejera-Segura et al., 2017**(10)	401	J774	BODIPY	yes	plasma	4 hours	t0	NA	NA	NA	NA
**Kopecky et al., 2017**(11)	1147	THP-1	3H	-	plasma	5 hours	per-well	NaOH	5.4%	7.9%	NA
**Koekemoer et al., 2017**(12)	1988	J774	3H	yes	serum	4 hours	t0	NA	4.7%	6.3%	NA
**Li et al., 2013**(13)	1150 +577	RAW264.7	3H or 14C	yes	serum	4 hours	per-well	hex isoprop	3.1-4.5%	7.6-11.4%	NA
**Liu et al., 2016**(14)	1737	J774	BODIPY	yes	serum	4 hours	t0	NA	4.7%	8.4%	NA
**Low-Kam et al., 2018**(15)	5293	J774	3H	yes/no	plasma	4 hours	per-well	NaOH	6.2%	18.4%	>3%
**Ogura et al., 2016**(16)	227	J774	3H	no	serum	4 hours	t0	hex isoprop	NA	NA	NA
**Potočnjak et al., 2016**(17)	152	J774	3H	yes	serum	4 hours	t0	hex isoprop	7.1%	6.8%	NA
**Ritsch et al., 2020**(18)	2468	J774	3H	yes	serum	4 hours	t0	NA	NA	NA	NA
**Rohatgi et al., 2014**(19)	2924	J774	BODIPY	yes	plasma	4 hours	t0	NA	3.3%	7.4%	NA
**Saleheen et al., 2015**(20)	1745	J774	3H	yes	serum	4 hours	t0	hex isoprop	4.5%	7%	NA
**Shea et al. 2019**(21)	930 +814	THP-1	-	-	serum	6 hours	Cholesterol mass efflux capacity	-	4.6%	NA	NA
**Zhang et al., 2016**(22)	313	J774	3H	yes	serum	4 hours	per-well	NA	NA	NA	NA

THP-1: THP-1 monocytes differentiated into macrophages; NaOH: sodium hydroxide; hex isoprop: hexane isopropanol lipid extraction; NA: not available (i.e. not described in main part or supplemental material of the respective study); "-" indicates that it was not applicable for the respective protocol.

[a]Apolipoprotein B-depleted acceptor

Supplemental Table S2: Summary of the previously published CEC assay protocols listed in Supplemental Table S1.

	Number/total studies	% of total	CV details
**Label**			
BODIPY	3/20	15.0	
^{3}H	16/20	80.0	
None	1/20	5.0	
**Cell line**			
J774 macrophages	15/20	75.0	
THP-1 cells differentiated into macrophages	4/20	20.0	
RAW264.7 macrophages	1/20	5.0	
**cAMP treatment (if applicable)**			
Reported	15/16	93.8	
Yes	13/15	86.7	
No	2/15	13.3	
**Acceptor: apoB-depleted**			
Serum	13/20	65.0	
Plasma	7/20	35.0	
**Duration of efflux**			
Reported	18/20	90.0	
4 hours	14/18	77.8	
5 hours	1/18	5.6	
6 hours	2/18	11.1	
24 hours	1/18	5.6	
**Measurement method**			
Reported	18/20	90.0	
Per-well method	6/18	33.3	
T0 method	10/18	55.6	
Other	2/18	11.1	
**Lysis reagent (if applicable)**			
Reported	10/18	58.6	
Sodium hydroxide	4/10	40.0	
Hexane isopropanol extraction	6/10	60.0	
**Intra-assay CV**			
Reported	13/20	65.0	
Min-max reported			3.3-7.3%
**Inter-assay CV**			
Reported	11/20	55.0	
Min-max reported			6.3-18.4%
**Reduction of inter assay CV**			
Reported	1/20	5.0	
Reduction			>3% reduction in CV

Supplemental Table S3: Comparison of assay performance of CEC values normalized to the resazurin absorbance ratio and non-normalized CEC values.

	t0 method	Per-well method
Intra-assay CV in% resazurin-normalized CEC/non-normalized CEC[a]	5.27/5.28	5.02/4.48
Inter-assay CV PC1 in% resazurin-normalized CEC/non-normalized CEC	19.94/16.74	17.27/11.88
Inter-assay CV PC2 in% resazurin-normalized CEC/non-normalized CEC	14.89/16.34	13.24/11.55

PC: Positive control. [a]excluding rejected samples.

Supplemental Figure S1: Fluorescence remaining in the plate after lysis for 1 hour and overnight for cholic acid (CA), sodium hydroxide (NaOH) and Triton-X-100 (TX100).

Cells were seeded in a black tissue-treated 96-well plate with a clear bottom and stained with BODIPY-cholesterol as described in the methods. After equilibration, cells were washed with DPBS and lysed with the respective lysis reagent as indicated. Fluorescence intensity (FI) remaining in the plate was calculated with the FI before and after removal of the lysate. FI of unstained cells lysed with the respective lysis reagent and condition was subtracted. Each dot represents one technical replicate.

Experiment 1:

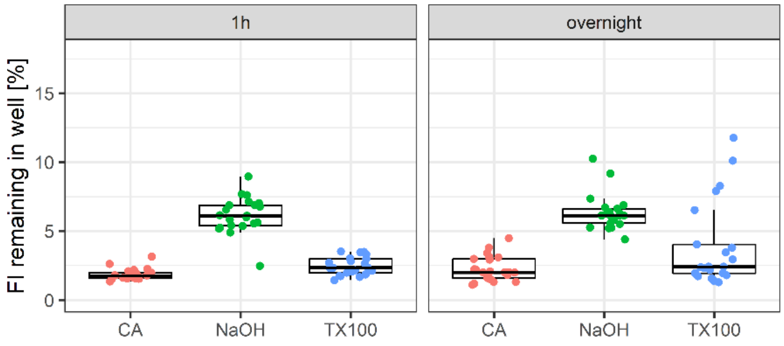

Experiment 2:

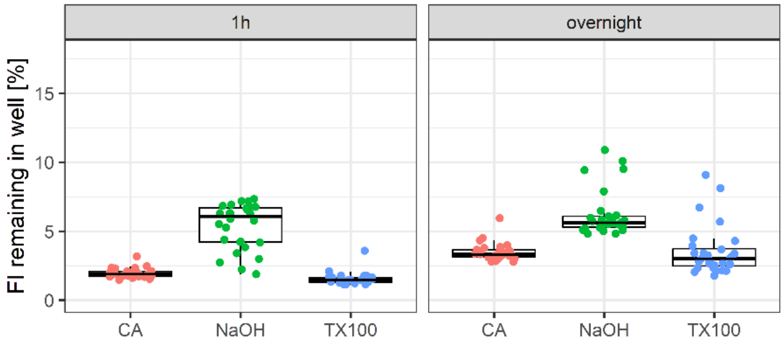

Experiment 3:

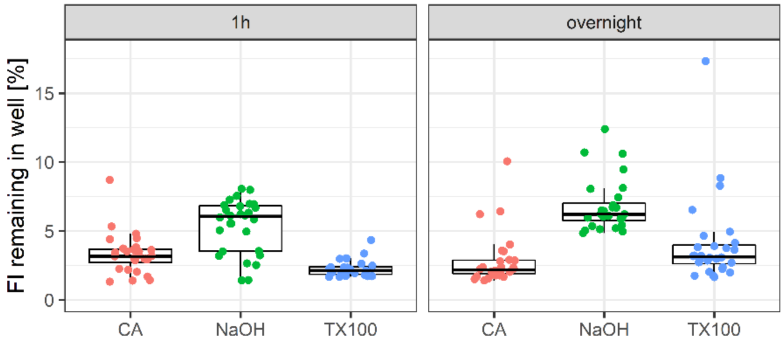

Supplemental Figure S2: Correlation of cell number with the resazurin absorbance ratio.

Dots and error bars represent mean and standard deviation from seven replicates. If no error bars are visible, they are contained within the symbol due to a very small variability of the measurements. The line represents the fitted linear regression line and the gray area around the line represents 95% confidence intervals. Cells were seeded, allowed to adhere for 4 hours and subsequently incubated with the resazurin assay for 3 hours.

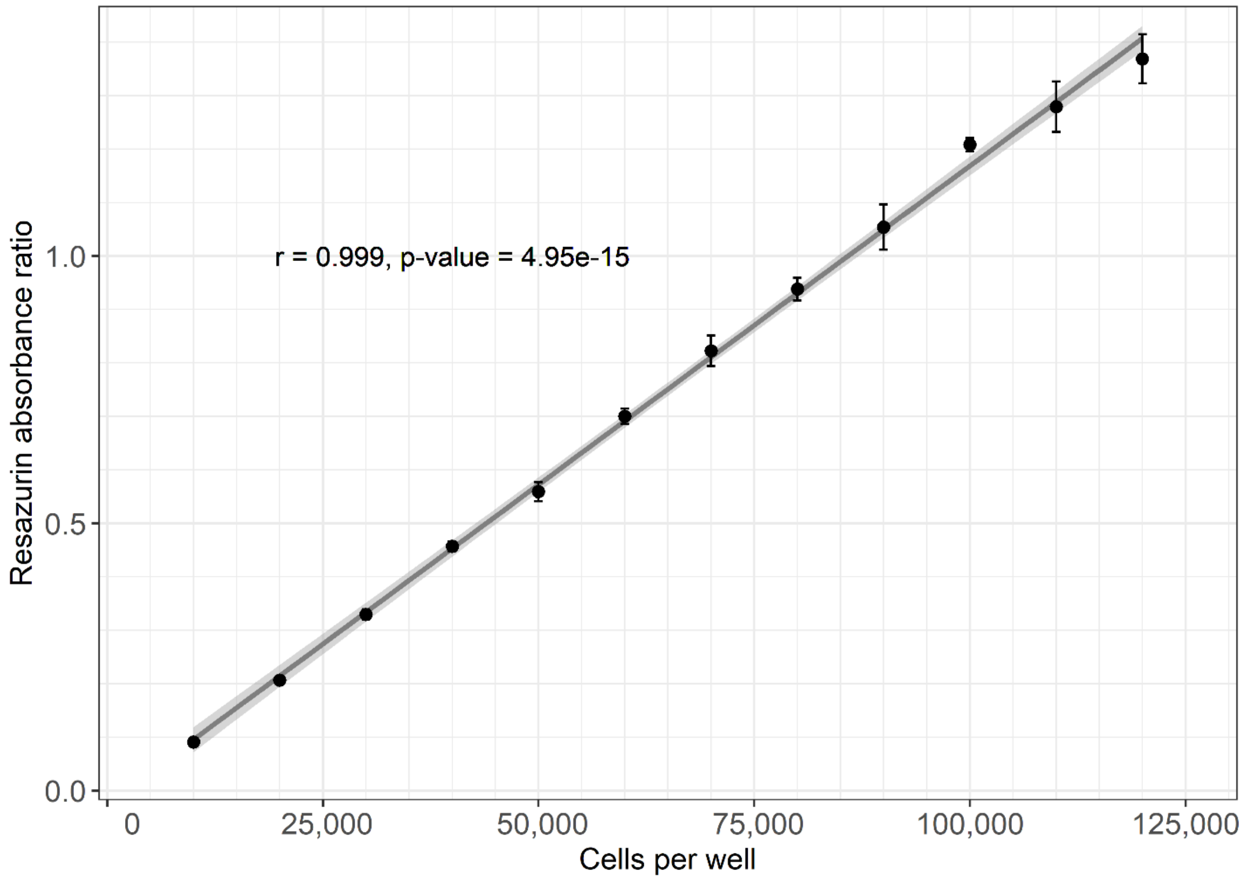

Supplemental Figure S3: Viability differs between seeded cell numbers.

Different cell numbers (35,000; 52,500; 70,000 [standard cell number for the CEC assay] and 105,000) were seeded per well and equilibrated as described for the CEC protocol. DNA of cells was stained with Hoechst 33342 (Thermo Fisher Scientific; 1:50,000) and dead cells with Acridine Orange/Propidium iodide (Logos Biosystems; 1:5,000) in RPMI-1640 without phenol red. Subsequently, cells were imaged on an Olympus BX61VS microscope and analyzed using the scanR high-content screening technology (Olympus). Cells were identified by Hoechst 33348 staining of their nuclei from the software, gated for single cells and viability was determined based on propidium iodide staining. A) Gating strategy. B) Proportion of dead single cells for different cell numbers. Each dot represents a technical replicate. The red bar represents the mean. Ns: not significant; *** p-value ≤ 0.0001.

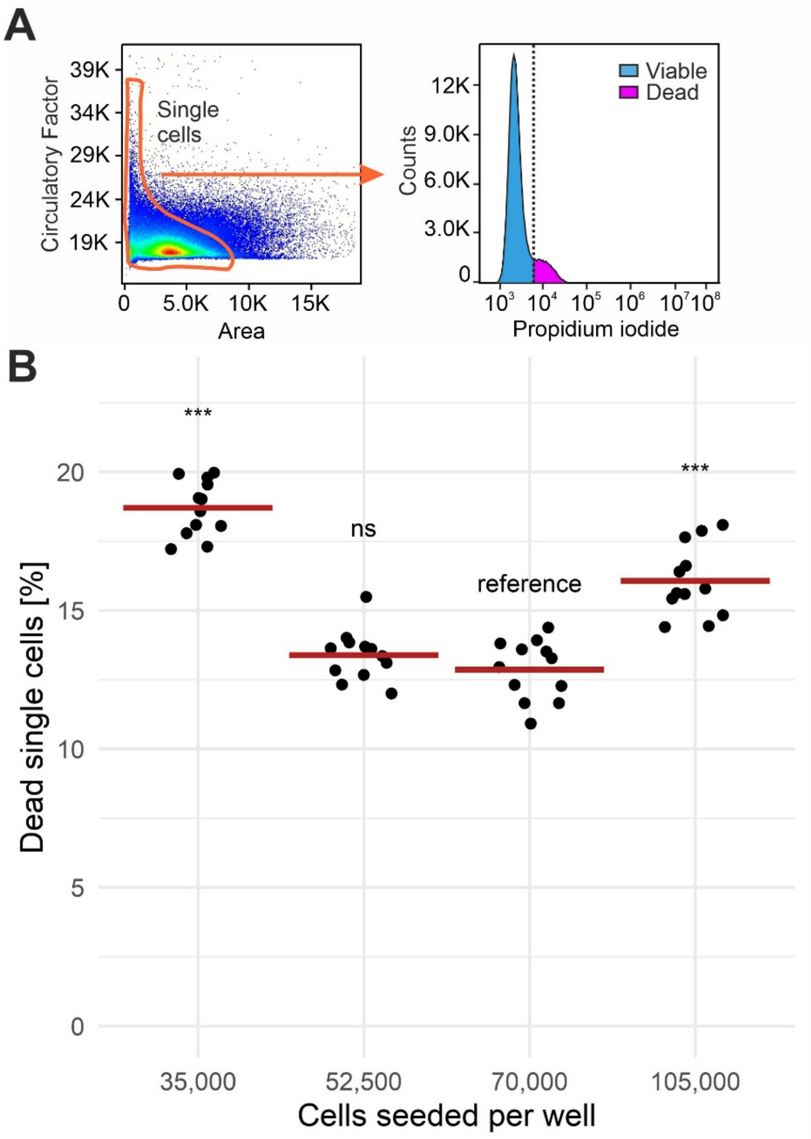

Supplemental Figure S4: For the per-well method, CEC from cAMP-treated cells is significantly higher than CEC from untreated cells (p=0.0006, n=5).

Dots and error bars represent mean ± standard deviation of triplicates. If no error bars are visible, they are contained within the symbol due to a very small variability of the measurements. S1-5: Sample 1-5.

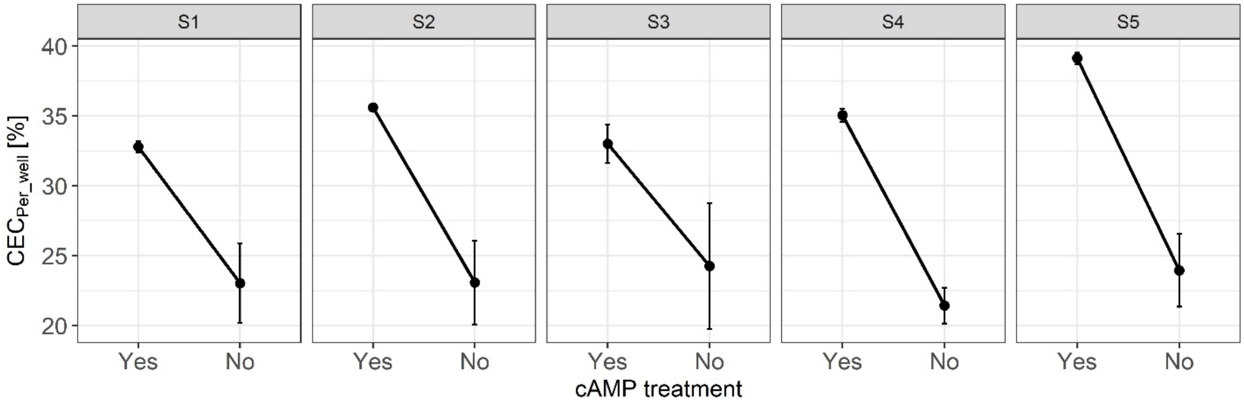

Supplemental Figure S5: For the t0 method, CEC from cAMP-treated cells is significantly higher than CEC from untreated cells (p=0.0015, n=5).

Dots and error bars represent mean ± standard deviation of triplicates. If no error bars are visible, they are contained within the symbol due to a very small variability of the measurements. S1-5: Sample 1-5.

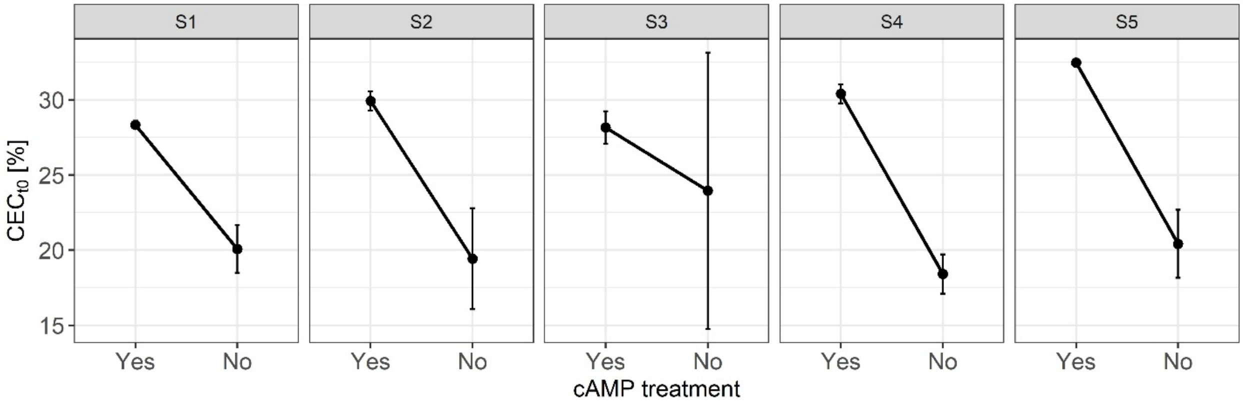

Supplemental Figure S6: CEC saturation curves for increasing amounts of apoB-depleted serum of five healthy individuals for the per-well method.

The gray line is fitted to a Michaelis-Menten model. Dots and error bars represent mean ± standard deviation of triplicates (of duplicates for S4). If no error bars are visible, they are contained within the symbol due to a very small variability of the measurements. S1-5: Sample 1-5.

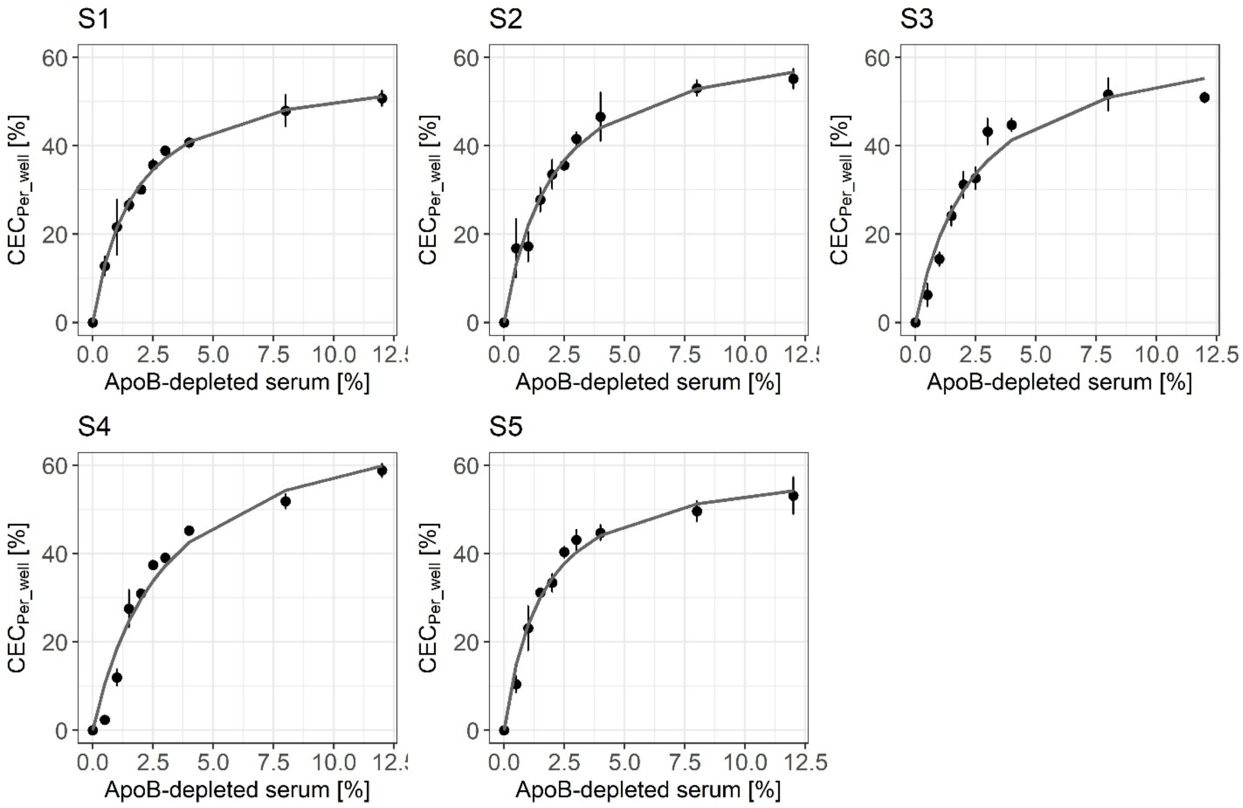

Supplemental Figure S7: CEC saturation curves for increasing amounts of apoB-depleted serum of five healthy individuals for the t0 method.

The gray line is fitted to a Michaelis-Menten model. Dots and error bars represent mean ± standard deviation of triplicates (of duplicates for S4). If no error bars are visible, they are contained within the symbol due to a very small variability of the measurements. S1-5: Sample 1-5.

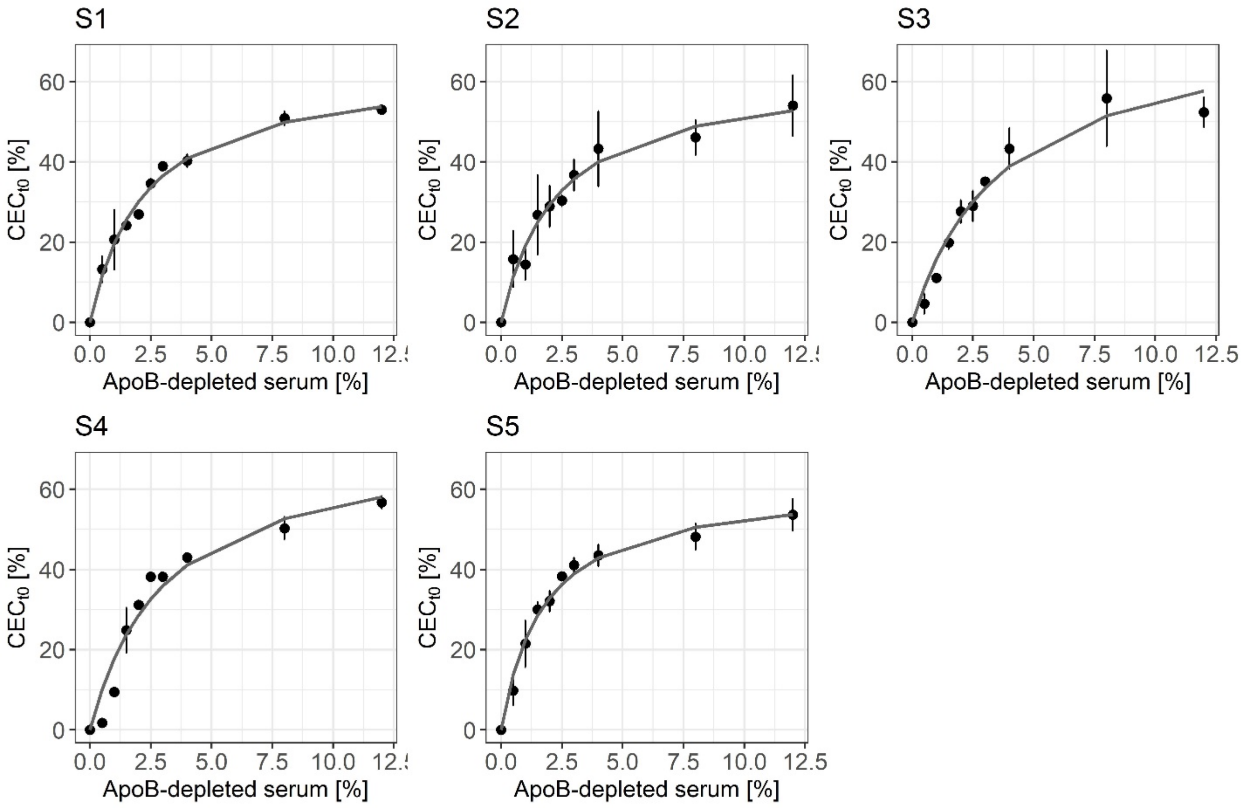

Supplemental Figure S8: Bland-Altman plots for CEC measured twice 25 days apart including samples with high CV.

(A) Uncorrected measurements for the t0 method, (B) uncorrected measurements for the per-well method, (C) corrected measurements for the t0 method and (D) corrected measurements for the per-well method. In total, 28 samples were measured twice but eight samples had to be rejected in the t0 calculation method due to high CV of replicates (marked in red). These eight samples would have fulfilled the quality control criteria for the per-well method except for one sample. Dashed lines represent mean ± 2 standard deviations of differences (95% limits of agreement). The gray solid line represents difference = 0. M1: measurement 1, M2: measurement 2.

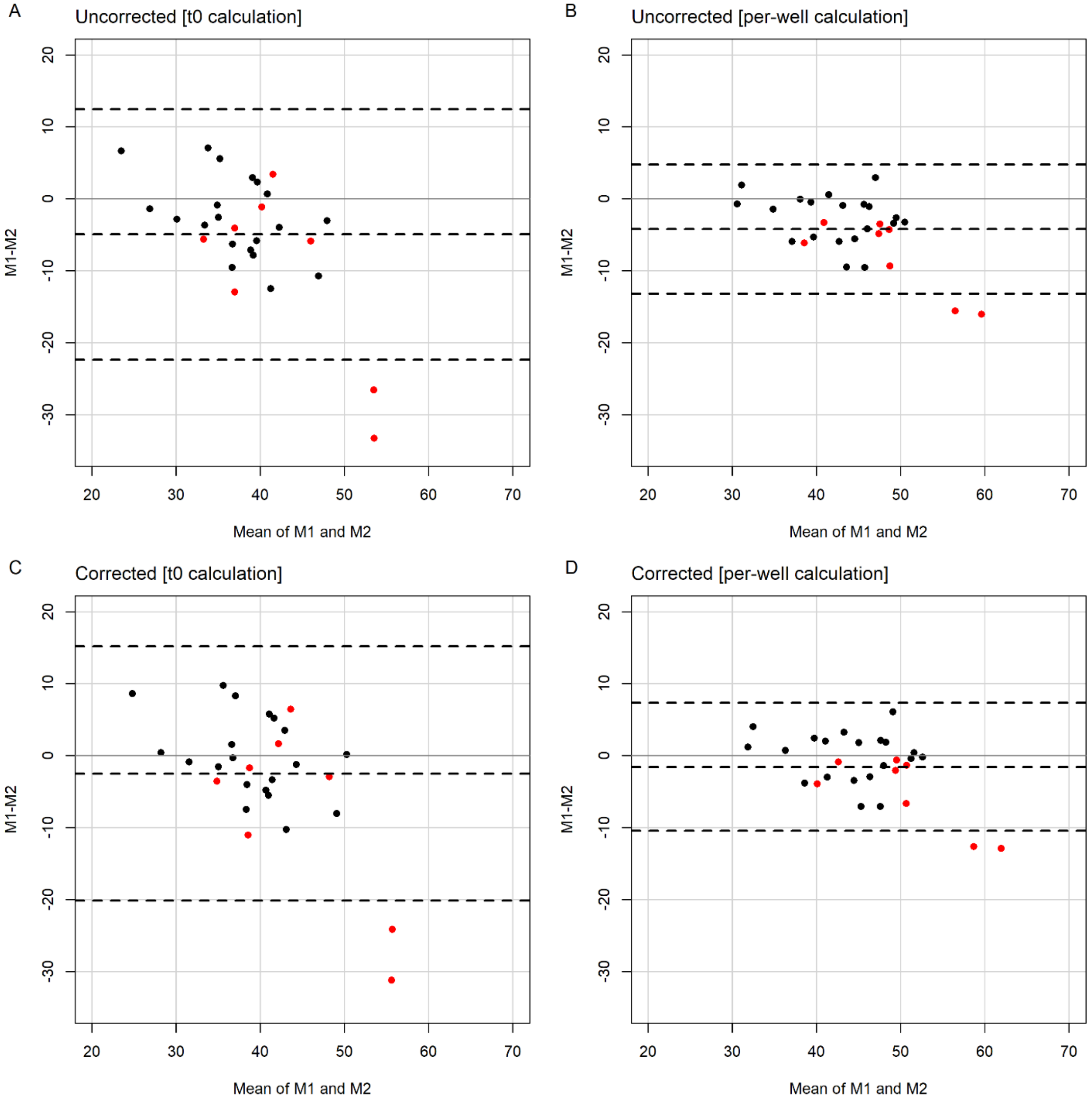

References

1. Qiu, C., X. Zhao, Q. Zhou, and Z. Zhang. 2017. High-density lipoprotein cholesterol efflux capacity is inversely associated with cardiovascular risk: A systematic review and meta-analysis. *Lipids Health Dis.* **16**: 1–11.

2. Soria-Florido, M. T., H. Schröder, M. Grau, M. Fitó, and C. Lassale. 2020. High density lipoprotein functionality and cardiovascular events and mortality: A systematic review and meta-analysis. *Atherosclerosis*. **302**: 36–42.

3. Annema, W., A. Dikkers, J. Freark de Boer, R. P. F. Dullaart, J.-S. F. Sanders, S. J. L. Bakker, and U. J. F. Tietge. 2016. HDL Cholesterol Efflux Predicts Graft Failure in Renal Transplant Recipients. *J. Am. Soc. Nephrol.* **27**: 595–603.

4. Annema, W., H. M. Willemsen, J. F. de Boer, A. Dikkers, M. van der Giet, W. Nieuwland, A. C. Muller Kobold, L. J. van Pelt, R. H. J. A. Slart, I. C. C. van der Horst, R. P. F. Dullaart, R. A. Tio, and U. J. F. Tietge. 2016. HDL function is impaired in acute myocardial infarction independent of plasma HDL cholesterol levels. *J. Clin. Lipidol.* **10**: 1318–1328.

5. Bauer, L., S. Kern, K. S. Rogacev, I. E. Emrich, A. Zawada, D. Fliser, A. Heinemann, G. H. Heine, and G. Marsche. 2017. HDL Cholesterol Efflux Capacity and Cardiovascular Events in Patients With Chronic Kidney Disease. *69*. **69**: 246–247.

6. Hunjadi, M., C. Lamina, P. Kahler, T. Bernscherer, J. Viikari, T. Lehtimäki, M. Kähönen, M. Hurme, M. Juonala, L. Taittonen, T. Laitinen, E. Jokinen, P. Tossavainen, N. Hutri-Kähönen, O. Raitakari, and A. Ritsch. 2020. HDL cholesterol efflux capacity is inversely associated with subclinical cardiovascular risk markers in young adults: The cardiovascular risk in Young Finns study. *Sci. Rep.* **10**: 1–12.

7. Ishikawa, T., M. Ayaori, H. Uto-Kondo, T. Nakajima, M. Mutoh, and K. Ikewaki. 2015. High-density lipoprotein cholesterol efflux capacity as a relevant predictor of atherosclerotic coronary disease. *Atherosclerosis*. **242**: 318–322.

8. Khera, A. V., M. Cuchel, M. de la Llera-Moya, A. Rodrigues, M. F. Burke, K. Jafri, B. C. French, J. A. Phillips, M. L. Mucksavage, R. L. Wilensky, E. R. Mohler, G. H. Rothblat, D. J. Rader, D. Ph, J. A. Phillips, D. Ph, M. L. Mucksavage, M. Sc, R. L. Wilensky, E. R. Mohler, G. H. Rothblat, D. Ph, and D. J. Rader. 2011. Cholesterol Efflux Capacity, High-Density Lipoprotein Function, and Atherosclerosis. *N. Engl. J. Med.* **364**: 127–135.

9. Khera, A. V., O. V. Demler, S. J. Adelman, H. L. Collins, R. J. Glynn, P. M. Ridker, D. J. Rader, and S. Mora. 2017. Cholesterol Efflux Capacity, High-Density Lipoprotein Particle Number, and Incident Cardiovascular Events: An Analysis from the JUPITER Trial (Justification for the Use of Statins in Prevention: An Intervention Trial Evaluating Rosuvastatin). *Circulation*. **135**: 2494–2504.

10. Tejera-Segura, B., M. Macía-Díaz, J. D. Machado, A. de Vera-González, J. A. García-Dopico, J. M. Olmos, J. L. Hernández, F. Díaz-González, M. A. González-Gay, and I. Ferraz-Amaro. 2017. HDL cholesterol efflux capacity in rheumatoid arthritis patients: contributing factors and relationship with subclinical atherosclerosis. *Arthritis Res. Ther.* **19**: 113.

11. Kopecky, C., S. Ebtehaj, B. Genser, C. Drechsler, V. Krane, M. Antlanger, J. J. Kovarik, C. C. Kaltenecker, M. Parvizi, C. Wanner, T. Weichhart, M. D. Säemann, and U. J. F. Tietge. 2017. HDL Cholesterol Efflux Does Not Predict Cardiovascular Risk in Hemodialysis

Patients. *J. Am. Soc. Nephrol.* **28**: 769–775.

12. Koekemoer, A. L., V. Codd, N. G. D. Masca, C. P. Nelson, M. D. Musameh, B. M. Kaess, C. Hengstenberg, D. J. Rader, and N. J. Samani. 2017. Large-Scale Analysis of Determinants, Stability, and Heritability of High-Density Lipoprotein Cholesterol Efflux Capacity. *Arterioscler. Thromb. Vasc. Biol.* **37**: 1956–1962.
13. Li, X. M., W. H. W. Tang, M. K. Mosior, Y. Huang, Y. Wu, W. Matter, V. Gao, D. Schmitt, J. A. DiDonato, E. A. Fisher, J. D. Smith, and S. L. Hazen. 2013. Paradoxical association of enhanced cholesterol efflux with increased incident cardiovascular risks. *Arterioscler. Thromb. Vasc. Biol.* **33**: 1696–1705.
14. Liu, C., Y. Zhang, D. Ding, X. Li, Y. Yang, Q. Li, Y. Zheng, D. Wang, and W. Ling. 2016. Cholesterol efflux capacity is an independent predictor of all-cause and cardiovascular mortality in patients with coronary artery disease: A prospective cohort study. *Atherosclerosis*. **249**: 116–124.
15. Low-Kam, C., D. Rhainds, K. S. Lo, A. Barhdadi, M. Boulé, S. Alem, V. Pedneault-Gagnon, E. Rhéaume, M. Dubé, D. Busseuil, R. A. Hegele, G. Lettre, and J. Tardif. 2018. Variants at the APOE /C1/C2/C4 Locus Modulate Cholesterol Efflux Capacity Independently of High-Density Lipoprotein Cholesterol. *J. Am. Heart Assoc.* **7**: e009545.
16. Ogura, M., M. Hori, and M. Harada-Shiba. 2016. Association between Cholesterol Efflux Capacity and Atherosclerotic Cardiovascular Disease in Patients with Familial Hypercholesterolemia. *Arterioscler. Thromb. Vasc. Biol.* **36**: 181–188.
17. Potočnjak, I., V. Degoricija, M. Trbušić, S. D. Terešak, B. Radulović, G. Pregartner, A. Berghold, B. Tiran, G. Marsche, and S. Frank. 2016. Metrics of High-Density Lipoprotein Function and Hospital Mortality in Acute Heart Failure Patients. *PLoS One*. **11**: e0157507.
18. Ritsch, A., A. Duerr, P. Kahler, M. Hunjadi, T. Stojakovic, G. Silbernagel, H. Scharnagl, M. E. Kleber, and W. März. 2020. Cholesterol Efflux Capacity and Cardiovascular Disease: The Ludwigshafen Risk and Cardiovascular Health (LURIC) Study. *Biomedicines*. **8**: 524.
19. Rohatgi, A., A. Khera, J. D. Berry, E. G. Givens, C. R. Ayers, K. E. Wedin, I. J. Neeland, I. S. Yuhanna, D. R. Rader, J. A. de Lemos, P. W. Shaul, D. Ph, I. J. Neeland, I. S. Yuhanna, D. Ph, D. R. Rader, J. A. De Lemos, and P. W. Shaul. 2014. HDL cholesterol efflux capacity and incident cardiovascular events. *N. Engl. J. Med.* **371**: 2383–2393.
20. Saleheen, D., R. Scott, S. Javad, W. Zhao, A. Rodrigues, A. Picataggi, D. Lukmanova, M. L. Mucksavage, R. Luben, J. Billheimer, J. J. P. Kastelein, S. M. Boekholdt, K.-T. Khaw, N. Wareham, and D. J. Rader. 2015. Association of HDL cholesterol efflux capacity with incident coronary heart disease events: a prospective case-control study. *Lancet Diabetes Endocrinol.* **3**: 507–513.
21. Shea, S., J. H. Stein, N. W. Jorgensen, R. L. McClelland, L. Tascau, S. Shrager, J. W. Heinecke, L. Yvan-Charvet, and A. R. Tall. 2019. Cholesterol Mass Efflux Capacity, Incident Cardiovascular Disease, and Progression of Carotid Plaque: The Multi-Ethnic Study of Atherosclerosis. *Arterioscler. Thromb. Vasc. Biol.* **39**: 89–96.
22. Zhang, J., J. Xu, J. Wang, C. Wu, Y. Xu, Y. Wang, F. Deng, Z. Wang, X. Chen, M. Wu, and Y. Chen. 2016. Prognostic Usefulness of Serum Cholesterol Efflux Capacity in Patients with Coronary Artery Disease. *Am. J. Cardiol.* **117**: 508–514.
